# Spontaneous symmetry breaking in plasmon lattice lasers

**DOI:** 10.1126/sciadv.adn2723

**Published:** 2024-07-05

**Authors:** Nelson de Gaay Fortman, Radoslaw Kolkowski, Debapriya Pal, Said R. K. Rodriguez, Peter Schall, A. Femius Koenderink

**Affiliations:** ^1^ Institute of Physics, University of Amsterdam, NL1098XH Amsterdam, The Netherlands.; ^2^ Department of Physics of Information in Matter and Center for Nanophotonics, NWO-I Institute AMOLF, Science Park 104, NL1098XG Amsterdam, The Netherlands.; ^3^ Department of Applied Physics, Aalto University, P.O. Box 13500, FI-00076 Aalto, Finland.

## Abstract

Spontaneous symmetry breaking (SSB) is key for our understanding of phase transitions and the spontaneous emergence of order. In this work, we report that, for a two-dimensional (2D) periodic metasurface with gain, SSB occurs in the lasing transition. We study diffractive hexagonal plasmon nanoparticle lattices, where the *K*-points in momentum space provide two modes that are degenerate in frequency and identically distributed in space. Using femtosecond pulses to energize the gain medium, we simultaneously capture single-shot real-space and Fourier-space images of laser emission. By combining Fourier and real space, we resolve the two order parameters for which symmetry breaking simultaneously occurs: spatial parity and *U*(1) (rotational) symmetry breaking, evident respectively as random relative mode amplitude and phase. The methodology reported in this work is generally applicable to 2D plasmonic and dielectric metasurfaces and opens numerous opportunities for the study of SSB and the emergence of spatial coherence in metaphotonics.

## Teaser

Spontaneous symmetry breaking leads to random relative amplitude and phase between the *K* and *K'* laser modes.

## INTRODUCTION

In nature, there exist many physical systems that can spontaneously and abruptly evolve into an asymmetric state. This phenomenon of spontaneous symmetry breaking (SSB) is observed, for instance, in phase transitions, such as in the emergence of ferromagnetism, superfluidity, superconductivity, and Bose-Einstein condensation. In photonics, SSB occurs, for instance, at the lasing transition, marked by the emergence of a randomly manifested phase, and in degenerate resonant systems imbued with gain or nonlinearity, where SSB expresses as a random imbalance in the population of the modes. SSB has recently been studied in evanescently coupled “photonic molecule” nanocavities (*1*, *2*), in microcavities with degeneracy between counter-propagating modes (whispering gallery mode systems), with either gain or a Kerr nonlinearity (*3*–*9*), and in exciton-polariton microcavities and microcavity lattices (*10*–*13*). There is a large interest in understanding and harnessing the mechanisms of SSB in photonics. First, this is because photonic systems are ideal to study SSB as a general phenomenon: Nonlinear photonic resonators allow one to synthesize many dynamical nonlinear differential equations with SSB behavior, and, in addition, one can measure system response with high precision over many orders of magnitude in time. Second, from the application viewpoint, SSB is envisioned as key to realizing ultrasmall flip-flop optical memories, optically controllable circulators and isolators, and all-optical switching (*8*, *14*, *15*).

Plasmon lattice lasers are periodically arranged lattices of plasmon nanoparticles (antennas) embedded in a gain waveguide slab. Plasmon lattice lasers were first reported in 2003 (*16*) and have since then been studied extensively (*17*–*27*). These systems are akin to distributed feedback (DFB) lasers (*28*), but the weak feedback mechanism of a dielectric Bragg grating is replaced by that of collective plasmon modes: The plasmon lattice enables long-range interactions (nanoparticle-scattering cross sections are often larger than the unit cell), and, through diffractive resonances that are delocalized over the lattice, this system generates strong features in the frequency-momentum (ω-*k*) space. This type of nonlocal resonance originates from the field of plasmonic surface lattice resonances. Recent work generalizes this to the so-called nonlocal metasurfaces of irregular periodic arrays with collective modes (*29*). The plasmon surface lattice resonances have high quality factors and strong plasmonic near fields. These unique properties provide strong feedback (*24*), exceptional robustness to disorder (*25*), and ultrafast gain dynamics (*18*). In the plasmon lattice system, one can tailor the unit cell resonance (controlled by antenna size and shape) and lattice symmetry at will, providing opportunities to study plasmon versions of a rich family of tight-binding Hamiltonians, such as honeycomb and kagome lattices with topological properties (*30*–*32*), bound states in the continuum modes (*33*), and exceptional points (*34*). These works fall into the broader context of two-dimensional (2D) photonic simulation systems of seminal Hamiltonians in solid-state physics: For instance, topological metasurfaces and photonic crystals can realize the quantum (spin) Hall effect (*35*), and active photonic lattices provide a route to study Parity-Time (PT)-symmetry breaking physics (*36*) and topological lasing (*37*–*39*).

In this work, we demonstrate SSB in hexagonal plasmon lattice lasers, using the intrinsic degeneracy of Bloch modes at the *K-*symmetry points in reciprocal space, which are defined as the corners of the first Brillouin zone. Lasing from these points has been reported before in photonic crystal and plasmonic lattice lasers (*22*, *40*). However, these works did not report SSB. In our newly developed methodology, we use a pulsed femtosecond laser as pump to bring the system to lasing in every single shot, and we simultaneously perform real-space and Fourier-space microscopy to map the relative intensity and phase of the lasing modes from shot to shot. We uncover that this system concurrently shows parity symmetry breaking, observable in the direction of light emission, and rotational, i.e., *U*(1) symmetry breaking, observable as a random choice of relative phase between the lasing modes (*41*). We reproduce this behavior with a minimal density matrix–based dynamic model, showing that the SSB origin can be attributed to the inherently stochastic noise in spontaneous emission that initiates the lasing. While conventionally studied coupled microcavity systems (*1*, *2*, *10*–*13*) are ideal for two-mode coupling or optical simulators of nonlinear Hamiltonians with just nearest-neighbor interactions, metasurfaces provide an even richer design space to construct symmetries, mode degeneracies, and long-range interactions. The extended 2D nature of the lattice laser also means that one can go beyond mapping only overall mode populations: Our microscopy methods resolve spatial structure in the SSB, giving direct insight into the spatial structure of the spontaneously emerging coherence. Therefore, this work opens a rich venue for studying SSB in photonics.

## RESULTS

**Fig. 1.**
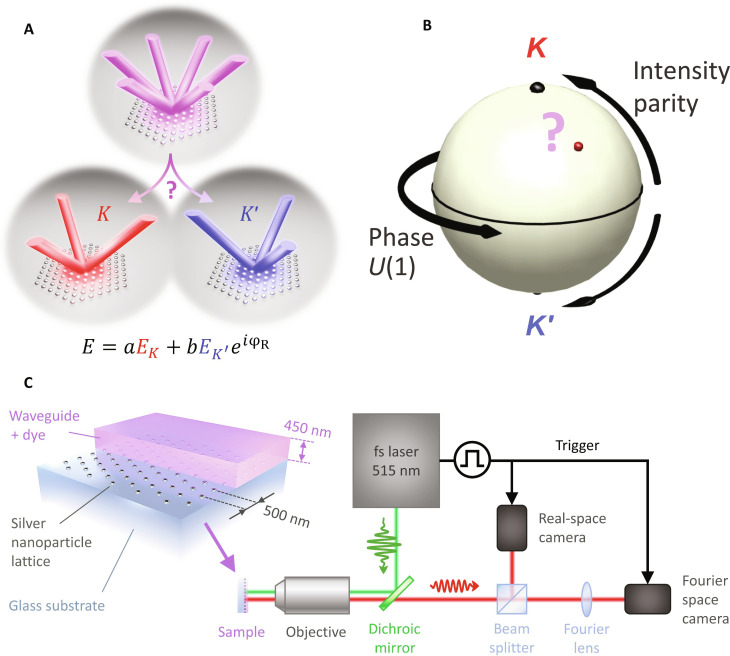
Single-shot microscopy retrieves relative intensity and phase of symmetry broken *K* and *K′* laser modes. (**A**) *K-*point lasing in hexagonal plasmon lattice lasers occurs on two decoupled modes, degenerate in frequency and space, and only differing in parity. Spontaneous symmetry breaking occurs in relative amplitude between the *K* and *K′* mode (parity breaking) and in relative phase [*U*(1) symmetry breaking]. (**B**) The phase space maps to the unit sphere, where the distance from the equator maps parity breaking, and the azimuth maps relative phase. (**C**) Plasmon lattices are embedded in a planar polymer waveguide with organic dye to provide gain. We study lasing in a high–numerical aperture (NA) microscope with single-shot real-space and Fourier imaging capabilities, synchronized to a 20-Hz train of pump pulses (515 nm, 250 fs).

Figure 1 illustrates our approach. We study hexagonal periodic lattices of plasmonic nanodisks (Ag, diameter of 80 nm, and height of 30 nm) embedded in a polymer waveguide doped with a laser dye (fabrication described in Materials and Methods). This layer acts both as a gain medium and as a planar waveguide with a single transverse electric (TE) and transverse magnetic (TM) mode. The dominantly in-plane nanoantenna polarizability makes this geometry especially favorable to TE-mode DFB lasing. The 500-nm lattice pitch creates a *K*-point lasing condition near 580-nm wavelength. The 2D hexagonal lattice provides degeneracy at the *K*-point both in frequency and in real space. The Brillouin zone has six *K-*points that fall apart in two decoupled sets (henceforth *K-* and *K′-*points), each consisting of three points that are internally connected by a reciprocal lattice vector. In a scalar description, at a given *K*-point, there exist three modes, two of which form a pair of doubly degenerate *E*-modes, and one is the remaining *A*_1_ mode, which is the only mode that forms a flat band (*42*) required for lasing. While group theory is more involved for polarized waves, the first-order TE waveguide mode still has the same separation into a degenerate doublet and one flat band (*43*). Not only the *K* and *K′* flat band modes are frequency degenerate, but also their local fields *E*_*K*,*K*′_(**r**) are exactly identical in terms of energy density (∣*E_K_*(**r**)∣^2 ^= ∣*E*_*K*′_(**r**)∣^2^). The only difference is in the mirrored wave vector content. This offers the condition for spontaneous parity symmetry breaking: lasing just on the *K* or just on the *K′* condition will correspond to three instead of six far-field output spots, with mirrored orientation (Fig. 1A). Lasing of both modes in some superposition will furthermore imply the spontaneous emergence of a random relative phase φ_R_, breaking *U*(1) symmetry (*44*). In this work, we visualize the phase space for such SSB on the unit sphere (Fig. 1B): Moving away from the equator represents parity breaking (pure *K* and *K′* lasing at the north and south poles, respectively), while azimuth represents the relative phase.

**Fig. 2.**
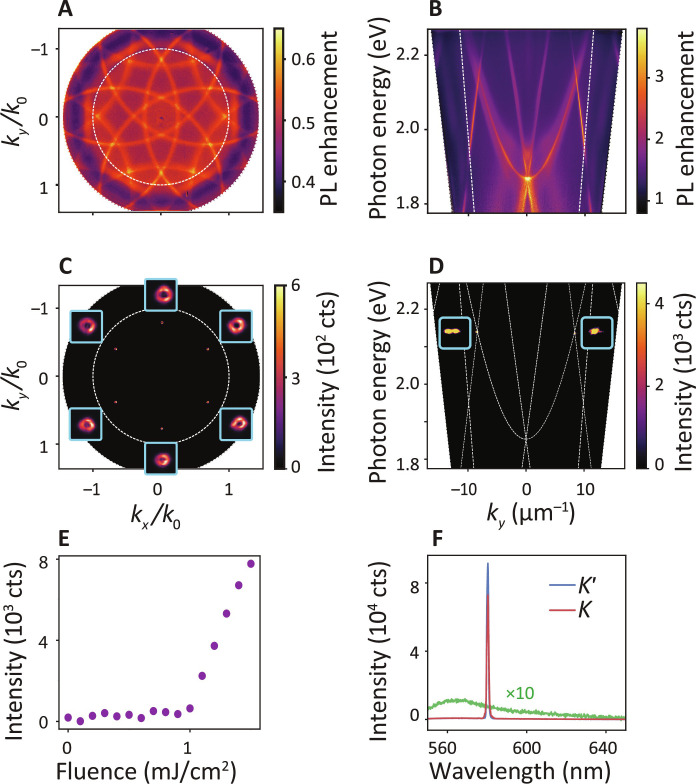
*K-*point lasing averaged over multiple laser shots. (**A**) Fourier image of photoluminescence (PL) shows the isofrequency contours of the guided mode dispersion curves that are repeated in the momentum space due to periodicity (below lasing threshold). Three-way crossings just within the NA = 1 circle (dashed) correspond to the *K-*points. (**B**) Below threshold PL band structure (dispersion image) showing *K-*point intersections at *ħ*ω = 2*.*15 eV. (**C**) Above-threshold Fourier image showing lasing at the six *K-*points in Fourier-space, with 5*×* enlarged beam spots as insets (averaged over 150 shots). The beam spots are doughnut-shaped due to the quasi-BIC nature of the lasing condition. (**D**) Lasing at the *K-*points in the dispersion image (six shots summed), with calculated free photon lines (insets show enlarged lasing spots). (**E**) Input-output power curve. (**F**) Above threshold, the broad fluorescence spectrum (green curve) narrows to two sharp lasing peaks (red and blue curves). The pump fluence of the lasing spectra is 3*×* larger than the threshold fluence. The emission wavelengths at *K-* and *K′*-points are identical. The values plotted in [(C) to (F)] are in units of camera counts (cts).

To map the symmetry breaking both in intensity parity and in phase, we use our newly developed simultaneous single-shot real- and Fourier-space microscopy method (Fig. 1C). The samples are pumped by 250-fs pulses at 515-nm wavelength. Emission collected by a high–numerical aperture (NA) microscope objective is split into two optical tracks, each with a camera synchronized with the 20-Hz laser pulse train. By imaging real space in one track, while inserting a Fourier lens in the other, we obtain statistics to correlate Fourier-space and real-space output over long sequences of single-shot experiments. To assess the dispersion relation underlying the laser behavior, we collect Fourier and dispersion (band structure) images of photoluminescence (PL) enhancement (Fig. 2, A and B) by pumping at low fluence yet high repetition rate (1 MHz). We refer to Materials and Methods for more details. The PL enhancement Fourier image (Fig. 2A) shows that exactly three circular bands cross at each *K*-point. These bands are the 2D slab waveguide modes folded by the lattice periodicity (*45*). The intersections are also clearly visible in the dispersion (Fig. 2B). The threefold degeneracy is consistent with the fact that *K-*point lasing corresponds to feedback on three reciprocal lattice vectors *G* that form a closed triangular loop (*40*). The dispersion image, taken by projecting the *k_x_* = 0 slice of the Fourier image on the entrance slit of a spectrometer, samples one of the *K′-*points (negative *k_y_*) and one of the *K-*points (positive *k_y_*), at an energy of 2.15 eV. From the dispersion image, we estimate the quality factor of the diffractive surface lattice resonance near the *K-*point as *Q* = 120.

To measure lasing, we operate in single-shot mode. Increasing the pump pulse energy leads to a sudden nonlinear increase in output power at the *K-*points together with a very clear spectral narrowing (Fig. 2, E and F). The thresholds of 1 mJ/cm^2^ (50-nJ pulse energy) are consistent with earlier works on plasmon lattice lasing (*22*, *24*, *27*). Fourier images integrated over many shots (Fig. 2C) distinctly show six high-intensity spots at the *K-*points. Each spot has a doughnut shape, reflecting the fact that the lasing mode is prevented from radiating directly into the far field due to its symmetry (a dark mode). This effect is known as a symmetry-protected optical bound state in the continuum (BIC) (*46*), where the DFB laser will choose as laser condition the band edge of lowest loss, which is the band that is dark precisely at the *K-*point, as opposed to a radiatively damped band. While this is a BIC in the infinite lattice, lasing will occur over a finite spatial envelope. This translates into a finite-width wave vector content around the *K-*points (*47*–*49*). In current nomenclature in the field, this is considered a quasi-BIC, where the finite extent opens the BIC to radiation (*46*, *50*). Last, the dispersion image for lasing is shown in Fig. 2D. Two intensity peaks from lasing, at the *K-* and *K′-*points, occur at the same wavelength (Fig. 2F), highlighting the frequency degeneracy that the hexagonal lattice symmetry guarantees for *K*/*K′* modes. The imbalance in intensity between the two spectra is due to experimental reasons that are explained in the Supplementary Materials.

**Fig. 3.**
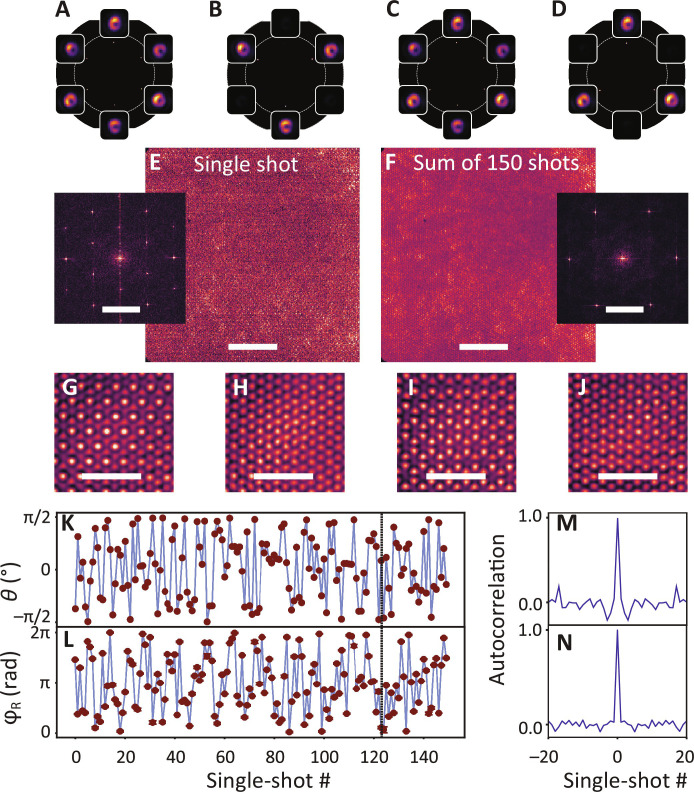
Single-shot *K-*point lasing and SSB in Fourier space and real space. (**A** to **D**) Single-shot Fourier images taken from a single experiment run. Shots 1 to 3 [(A) to (C)] are directly subsequent to each other. [(B) and (D)] Parity breaking toward pure *K*/*K′*-point lasing; [(A) and (C)] cases of equal superposition. (**E** and **F**) Single-shot and ensemble-averaged real-space images (scale bars, 10 μm). Insets show 2D Fourier transforms (scale bars, 10 μm^*−*1^). (**G** to **J**) Fourier-filtered close-ups of the real-space images (scale bars, 3 μm), corresponding to the Fourier images [(A) and (B)]. (**K** and **L**) Sequence of relative amplitude (parity breaking) and phase [*U*(1) symmetry breaking] for a 150-shot data sequence. The dashed line highlights the location of shots [(A) to (C)]. (**M** and **N**) Temporal autocorrelations of the data in (K) and (L). The traces are taken with a pump power of 1.8 mJ/cm^2^.

SSB in parity is directly evident in sequences of single-shot Fourier images. Figure 3 (A to D) shows four single-shot images taken from a run of several hundred laser shots. For each of the six *K-*point lasing spots, we observe very large shot-to-shot differences in intensity. Figure 3 (A to C) shows three directly subsequent shots, in the first of which both *K-* and *K′*-points appear equally bright, while the next frame has hardly any *K′*-point emission (only three lasing spots at the *K-*points). The opposite (parity breaking entirely toward *K′*-point lasing) is evident in Fig. 3D. In the entire data sequence, no spot patterns occur except linear superpositions of the *K* and *K′* triplets. Given the observation that the lasing output is intermittent with a different intensity ratio of *K* and *K′* modes in each shot, a relevant question is what the relative phase between the two modes is in a given realization, and how that phase is statistically distributed over all shots. Because the *K* and *K′* modes have no overlap in the Fourier space, Fourier imaging cannot reveal relative phase. However, the relative phase can be determined from single-shot real-space images, which we acquire simultaneously with Fourier images. Upon crossing the lasing threshold, the real-space appearance of the sample transitions from the diffuse glow of fluorescence to a structured speckled pattern that directly evidences the emergence of both spatial and temporal coherence (*25*). Figure 3E shows a typical single-shot real-space image over an area of 44 μm by 44 μm, while Fig. 3F shows the sum over many laser shots. In both results, the hexagonal lattice symmetry is visible. However, 2D Fourier transforms (insets) reveal a marked difference. The ensemble-averaged image only shows Fourier components commensurate with the lattice of particles (six sharp peaks in the Fourier-transformed images). The single-shot image instead shows additional spatial structure, evident as a six-tuple of Fourier peaks at wave vectors shorter by a factor $\sqrt{3}$. These peaks point toward the emergence of longer-range periodicities. Motivated by this observation, we apply Fourier-domain filtering (explained in the Supplementary Materials) and examine close-ups of Fourier-filtered real-space data. Real space images in Figure 3 (G to J) correspond directly to the single-shot Fourier images in Fig. 3 (A to D). Whenever only *K* (shot 2) or only *K′* (shot 4) lasing occurs, the intensity patterns are simply hexagonal with periodicity identical to the particle lattice. In stark contrast, when both *K-* and *K′*-points substantially lase (shots 1 and 3), we observe a honeycomb supercell pattern. Moreover, while shots 1 and 3 have very similar Fourier images, the spatial patterns are distinct: The shot 3 honeycomb pattern is clearly shifted horizontally and vertically compared to shot 1. This variation in spatial pattern encodes the random relative phase between *K-* and *K′*-point lasing.

To explain how we retrieve relative phase from real-space images, we consider a simple scalar coupled-mode model for *K*/*K′*-point Bloch modes (*51*, *52*). The *A*_1_ mode at the *K-*point is a coherent superposition of three plane waves of identical amplitude and phase, with in-plane momentum given by the three *K-*points

$$E_{K}\left(r\right)=e^{iK_{1}\cdot r}+e^{iK_{2}\cdot r}+e^{iK_{3}\cdot r}$$ (1)

The *K′* field is described by the same equation but substituting the *K′*-points as in-plane momenta. For both of these modes individually, the local intensity distributions correspond to identical simple hexagonal patterns with peak intensities at the positions of lattice nodes. However, for superpositions *E_T_*(**r**) = *aE_K_*(**r**) + *bE*_*K*′_(**r**)*e*^*i*φ_R_^, (subscript T for total field) the intensity distributions acquire a superlattice periodicity that is $\sqrt{3}$ times larger than the original lattice pitch. The relative amplitudes *a* and *b* affect the contrast, but not the topology of these honeycomb intensity patterns. The alignment of the superlattice intensity pattern relative to the underlying particle lattice depends on the relative phase φ_R_ (see the Supplementary Materials for a plot catalogue). The simple coupled-mode model qualitatively rationalizes all our observations, namely, (i) whenever lasing is purely on the *K-* or *K′*-point (shots 2 and 4), the intensity distribution is simply hexagonal. The real-space intensity distributions are identical, although the far-field directions of lasing emission are mirrored; (ii) honeycomb patterns occur when *K* and *K′* modes both lase, and the observed spatial shift from frame to frame can be understood as shot-to-shot variations in relative phase φ_R_ (shots 1 and 3 in Fig. 3); (iii) the incoherent sum over all frames washes out this interference and is simply hexagonal (Fig. 3F). To extract the relative phase φ_R_ for each laser shot, we take a small section of the real-space data (as selected for Fig. 3, G to J) and fit the coupled mode model, where the relative amplitude is not a free parameter but fixed by the Fourier images (see the Supplementary Materials for methodology).

Figure 3 (K and L) reports on the extracted fluctuations in parity breaking (*K*/*K′* contrast) and relative phase (random phase) in a typical measurement run. In line with Fig. 1B, we quantify the *K*/*K′* intensity contrast as an angle θ defined for intensity *I_K_* in the *K* modes and *I*_*K*′_ in the *K′* modes as tan(θ/2) = (*I_K_* − *I*_*K*′_)/(*I_K_* + *I*_*K*′_). From the time traces, we conclude that both the intensity ratio and relative phase manifest randomly and independently from shot to shot. The normalized temporal autocorrelations (Fig. 3, M and N) for both the *K*/*K′* intensity ratio and the relative phase show no correlation beyond ∆*n* = 0. This observation is consistent with the notion that the symmetry breaking is not from any explicit/geometric symmetry breaking but is purely spontaneous: It arises from the inherently stochastic fluctuations in the spontaneous emission seed that initiates lasing. In the system at hand, beyond simply being degenerate in spectrum and energy density ∣*E_K_*(**r**)∣^2^, the *K* and *K′* modes are even fully identical up to complex conjugation *E_K_*(**r**) *= E*_*K*′_(**r**)^*^, physically meaning time reversal. This means that overlap integrals ⟨*f*∣∆∣*f*⟩ with geometric perturbations, say in dielectric constant ∆ϵ, waveguide height ∆*h*, or particle polarizability ∆α, tend to be identical for both modes, especially for predominantly real-valued perturbations. These degeneracies imply deep robustness against geometric disorder in the sample and pump field. For instance, in our experiment, we were unable to impart explicit symmetry breaking with linear pump polarization, although the pump field does have near-field hot spots that rotate with input polarization. To conclude, this system shows pure SSB, which is a result of its inherent protection from explicit symmetry breaking caused by an inhomogeneous gain distribution. Therefore, we argue that this system stands out as a platform for exploring SSB in the lasing transition, as compared with systems that do not enjoy this degree of protection [for instance, coupled cavity lasers; (*1*)].

**Fig. 4.**
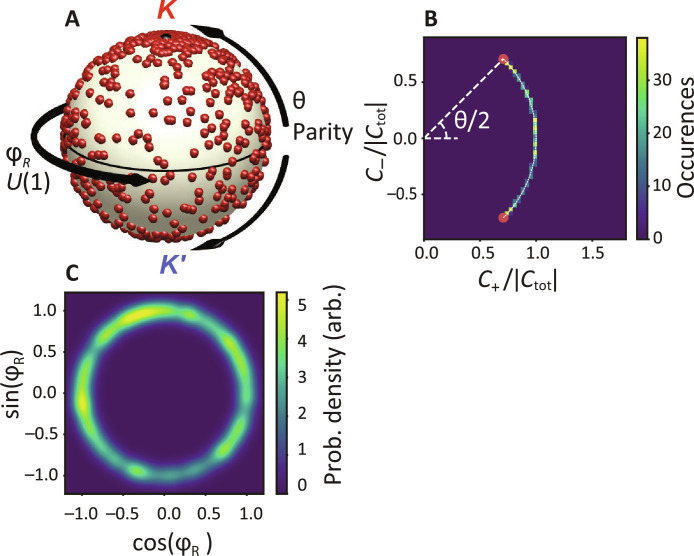
SSB order parameters θ and φ_R_, mapped onto the surface of a unit sphere. (**A**) SSB phase space samples in a 900-shot measurement run. Each shot is plotted as a data point on the unit sphere according to its relative phase (azimuth φ_R_) and relative amplitude (parity breaking, distance θ from equator). (**B** and **C**) Histograms of the parity breaking θ and random phase φ_R_.

To visualize how the SSB order parameters of this plasmon lattice laser distribute across the phase space, we consider the distribution of the intensity contrast parameter θ and the phase φ_R_ in Fig. 4. For a run of 900 shots, we place each shot on the unit sphere in Fig. 4A, while Fig. 4 (B and C) present the same results in the form of histograms for each parameter individually. With regard to the parity breaking, we construct a histogram by a projection method that not only takes into account the *K*/*K′* contrast but that also reports on whether spot patterns are strict superpositions of just *K*/*K′* lasing (see Materials and Methods). In short, Fig. 4B reads as follows: If all six lasing spots are equally bright, then *c*_+_ = 1 and *c*_−_ = 0. If instead lasing emission is just in the *K* (respectively, *K′*) directions, then $c_{+}=\pm c_{-}=\sqrt{1/2}$ . Any other linear combination of pure *K-* and *K′*-point lasing will result in a plot coordinate on the circular arc connecting these extremes and at angle θ*/*2 relative to the *x* axis. Last, any hypothetical intensity pattern that is not a linear superposition of *K-* and *K′*-point lasing (e.g., if just a single laser spot would appear) will result in a coordinate interior to the circular arc. The histogram evidences that no such patterns occur, verifying that all laser shots are true linear combinations of *K-* and *K′*-point lasing. Analysis as function of pump power (see the Supplementary Materials) shows that the histogram is identical irrespective of how high above threshold one operates. To test whether the variation in contrast θ samples the entire interval from *−*π*/*2 to π*/*2 uniformly, we subject this dataset to the Kolmogorov-Smirnov (KS) test to find whether the data are likely drawn from a uniform distribution. For the set of 150 data frames taken at the highest power (1.8 mJ/cm^2^), we calculate a KS value of 0.054, evidencing that the null hypothesis at α = 5% holds. While some runs marginally fail the test at this evel of strictness, we view that, quantitatively, the data are in good accord with a uniform distribution in θ. For details, refer to the Supplementary Materials.

This expression of symmetry breaking is comparable to recent results on orbital angular momentum microlasers (*9*) and different from the more usually reported bistability in which a system switches randomly to just one of the initially degenerate modes (*1*, *2*). One can ask whether this difference arises from the pulsed nature of our experiment, as previous studies on bistability either used quasi–continuous-wave conditions or pulses substantially longer than the intrinsic gain dynamics. To verify whether the pumping conditions affect the observed SSB character, we repeated the experiments with 500-ps instead of 250-fs pulses. These pulses are longer than the picosecond timescales associated with DFB laser dynamics. Despite pumping the system with such long pulses, we observe the same continuous histogram as in Fig. 4B (see Supplementary Materials). Furthermore, the fact that the real-space maps encode the relative phase rules out the possibility of rapid switching between the *K* and *K′* lasing within a single shot. Turning to the phase, Fig. 4C plots the probability density for observing (cosφ_R_, sinφ_R_). The relative phase between the *K* and *K′* is “picked” randomly as expected for *U*(1) symmetry breaking, where a system’s phase evolves as a second order phase transition from a highly symmetric (spontaneous emission) to locking in a single phase above lasing threshold (*41*, *44*). We also subject the φ_R_ data to the KS test, and we find that it generally passes the null hypothesis for strictness α = 5%. Last, we note that breaking the parity symmetry and *U*(1) symmetry leads to uncorrelated choices in θ and φ_R_ on the unit sphere. Although the data on the sphere suggest an accumulation of points close to the poles, this does not mean that θ is biased: Drawing θ and φ_R_ independently and uniformly is not equivalent to picking points uniformly over the unit sphere (due to the sin θ Jacobian).

We theoretically substantiate the special SSB phase space with a density matrix–based dynamic model, adapted from (*39*) and with parameters pertinent to our experiment. This minimal model (explained in detail in the Supplementary Materials) reproduces similar SSB behavior (fig. S10C) as the femtosecond-pulsed experiment (Fig. 4A). In contrast, for continuous-wave pumping, the model shows full bifurcation in intensity between *K* and *K′*, similar to SSB in coupled cavity lasers. In this regime, the physics may even be richer: Analysis of truly degenerate semiconductor traveling-wave ring lasing shows that bistability, multistability, and oscillating alternation between attractors may occur for the case of truly degenerate modes that are each other’s time-reversed companion (*5*). This exemplifies the rich and challenging field that our work opens up, both for experiments and theory.

## DISCUSSION

In summary, we observe SSB in *K-*point plasmon lattice lasers, where the symmetry is broken both in relative amplitude and phase between the two (*K* and *K′*) lasing modes. The SSB in intensity implies a breaking of parity symmetry, while the SSB in phase is a *U*(1) symmetry breaking. A key aspect of our work is the methodology of simultaneous single-shot Fourier- and real-space imaging, which should be contrasted to the earlier time-averaged measurements. This methodology is generally applicable to a large library of metasurface lasers with photonic lattice systems in which one can design symmetries and degeneracies at will (e.g., honeycomb, kagome lattices, and topological photonic crystals) (*23*, *31*, *46*, *53*). Therefore, our work opens important opportunities in the study of SSB and emergence of spatial coherence.

An example of the important role of symmetry and degeneracy can already be seen in hexagonal lattices, where one can compare *M*-point and *K-*point lasing (*54*). For *K-*point lasing, the lattice symmetry rigorously guarantees that modes are exactly co-localized in space and exactly degenerate in energy. *M*-point lasing is also associated to six output spots but, instead, arises from three distinct modes, each engaging two *M*-points. These modes are degenerate in frequency but distinct in space. Consequently, in the *M*-point laser, we observe three-way SSB that is easily turned into explicit symmetry breaking by changing the pump-field polarization and thereby the mode overlap with the near-field gain distribution. In this way, we can force lasing in just one out of the three modes. In contrast, we were unable to coerce *K-*point lasing into either the *K* or *K′* mode by changing pump geometry, reflecting the profound protection that the time-reversed modes experience against explicitly imparted disorder.

Another important perspective is to study the spontaneous emergence of coherence in space. Our methods image spatial variations of the order parameters: a degree of freedom that is not present in simple coupled cavity realizations of SSB. In the reported experiment, we can, for instance, fit real-space patterns in boxes of just a few unit cells and thereby assemble phase maps over the full microscope field of view (see the Supplementary Materials). In our system, such maps show mean-squared phase excursions of order 0.2 radians relative to the randomly chosen mean phase, with spatial correlation lengths that are of order of 5 μm. While the precise microscopic origin is out of the scope of this paper, these variations report in part on slight geometrical sample variations and, in part, arise from the DFB lasing physics. According to Kogelnik and Shank (*55*), even almost index-matched polymer DFB lasers show a rich spatial structure that is determined by interplay of the system band structure, the balance between gain, loss, and overall laser size (*27*). It is completely open how SSB in (nonlocal) DFB laser systems generalizes to the rich variety of nonlocal photonic systems that are now emerging, encompassing plasmonic and dielectric metasurface lasers, photonic crystal lasers, quasi-bound states in the continuum, and topological lasers, as well as systems that show photon or exciton-polariton Bose-Einstein condensate physics (*10*, *50*, *56*, *57*). Overall, our results demonstrate a practical route to quantifying the emergence of coherence in this entire array of photonic systems. Related to the above, the collective nonlocal resonances that are typical of the plasmon lattice laser turn out to be a cornerstone in the field of nonlocal metasurfaces (*29*). Therefore, we envision that our methods directly apply to this field, and we foresee interesting possibilities for spatially extended manipulation of the symmetry breaking in irregular periodic arrays.

Last, SSB in photonics can be associated with various applications. SSB in metasurfaces can be continuously distributed in intensity, as in this work, or can be multistable with two or more fixed points in phase space. Also, our observation that *M*-point lasers with SBB can be biased at will by pump polarization (as opposed to *K*-point lasers) shows that the multi-stability can be externally controlled. Such controllable bi- and multi-stability is a potential resource for photonic flip-flop memories. Lasers with chaotic output are also pursued as potentially high-bit rate, or triggerable, hardware random number generators (*58*). In this spirit, one can also envision SSB in metasurfaces as a resource for spiking artificial neural networks. Last, we view the methods developed in our work as laying the foundations for exploration of broken time symmetries, phase transitions, and handedness selectivity in metasurfaces coupled to active matter, such as gain media, 2D materials with valleytronic degrees of freedom, and in scenarios of strong coupling with excitons.

## MATERIALS AND METHODS

### Sample fabrication

We use 170-μm-thick microscope cover slips (Menzel) as substrates. A 150-nm layer of polymethyl methacrylate (PMMA) is deposited on the substrate by spin coating after cleaning them with base piranha. Then, we cover the PMMA with a thermally evaporated layer of 20-nm Ge, which acts as an etch mask for reactive ion etching after resist development. Over the Ge layer, we spin-coat a 65-nm layer of positive Allresist CSAR 62 resist in which we write the structures using electron beam lithography (approximate dose of 130 μC/cm^2^). The pattern is a hexagonal lattice of 500-nm period, consisting of 80-nm-diameter particles. After exposure, we develop the samples for 60 s in pentyl-acetate, 6 s in *O*-xylene and lastly 15 s in methyl isobutyl ketone with isopropyl alcohol (MIBK:IPA in 9:1 ratio). We then etch through the Ge and PMMA layer by reactive ion etching. Last, we evaporate a 30-nm Ag layer on the sample and perform liftoff in a 60*°*C acetone bath. This three-layer recipe from (*59*) has the benefit of a high resolution, defined by the thin CSAR layer, and yet a thick resist stack with high undercut for the liftoff. After fabrication of the plasmon lattice, we spin-coat a 450-nm-thick layer of MicroChem SU-8 3005 doped with rhodamine 6G (10 mg of Rh6G in 3 ml of cyclopentanone mixed with 1 ml of SU-8 or 0.5 wt % Rh6G) on the sample, as in (*24*). The polymer refractive index is estimated at 1.6, leading to a waveguide mode index of 1.55. On basis of variable-stripe-length measurements on unpatterned films, we estimate gain coefficients from 20 to 80 cm^*−*1^ over the relevant pump power range.

### Optical setup

As the light source for pumping our plasmon lattice laser, we use the frequency-doubled output from a Light Conversion Pharos laser, providing 515-nm wavelength and 250-fs pulse duration. The electronic signal of the internal pulse picker is used to drive single-shot camera measurements. We direct the pump light through an epilens and a microscope objective (Nikon CFI Plan Apochromat lambda 100*×*, NA = 1.45), to obtain a 70-μm-diameter collinear beam in the sample plane. The pump power is controlled by a motorized half-wave plate placed before a linear polarizer. The pump is filtered by a combination of a 532-nm dichroic mirror and a 550-nm long-pass filter. We image the sample plane with the same objective onto a camera chip (Bassler acA1920-40um) for real-space imaging. A beam splitter directs 50% of the light to another camera (Thorlabs CS2100M-USB) for Fourier-space imaging; in this track, we placed a Fourier lens in focus with the back focal plane of the objective via a 1:1 telescope. For spectrally resolved Fourier images (i.e., band structures), we direct the Fourier image through the entrance slit of a spectrometer (Andor Shamrock 163i), to which we mounted a camera (Ximea MC124MG-SY-UB). For single-shot lasing experiments, we use a repetition rate of 20 Hz and synchronize both the Bassler and Thorlabs camera to the laser to enable simultaneous single-shot imaging in real space and in Fourier space. To obtain band diagrams in (*k_x_*, *k_y_*) space or in (*k_y_*, ω) space, we operate in multi-shot mode with a repetition rate of 1 MHz and at low incident pulse power, thereby collecting below-threshold fluorescence. For the Fourier images below threshold, we insert a 550-nm (40-nm) bandpass filter to select a narrow band of emission wavelengths near the lasing conditions.

### *K*-point basis projection method

We convert the sequence of images into a time series of integrated intensities at each of the six lasing output spots, obtained by summing regions of interest (20 pixels across) around each spot. Having reduced the intensity information in the images to a vector of six intensities for each frame, we determine the projection of this six-vector onto the subspace of lasing in superpositions of the *K* and the *K′* modes. Let *I_N_* be the six-vector of intensities in frame *N* normalized to be of unit length, where the vector elements list intensities in the consecutive *K-*points enumerated in clockwise order, starting from the top spot. We determine the coefficients *c*_*N*,+_ = ⟨*I_N_*∣*v*_+_⟩ and *c*_*N*,−_ = ⟨*I_N_*∣*v*_−_⟩, where $v_{\pm }=1/\sqrt{6}\left(1,\pm \;1,1,\pm \;1,1,\pm \;1\right)$ , and ⟨.∣.⟩ stands for the inner product. We normalize to ⟨*I_N_*∣*I_N_*⟩. The normalized temporal autocorrelation for a parameter *x* is defined as $\text{Mean}\left\{\left[x\left(n+\text{∆}n\right)-\bar{x}\right]\left[x\left(n\right)-\bar{x}\right]\right\}/\text{Var}\left[x\right]$ where ∆*n* refers to comparing the time series at ∆*n* shots apart.

## Supplementary Materials

### This PDF file includes:

Sections S1 to S8 Figs. S1 to S10 Table S1

## References

[1] P. Hamel, S. Haddadi, F. Raineri, P. Monnier, G. Beaudoin, I. Sagnes, A. Levenson, A. M. Yacomotti, Spontaneous mirror-symmetry breaking in coupled photonic-crystal nanolasers. Nat. Photon. 9, 311–315 (2015).

[2] B. Garbin, A. Giraldo, K. J. H. Peters, N. G. R. Broderick, A. Spakman, F. Raineri, A. Levenson, S. R. K. Rodriguez, B. Krauskopf, A. M. Yacomotti, Spontaneous symmetry breaking in a coherently driven nanophotonic Bose-Hubbard dimer. Phys. Rev. Lett. 128, 053901 (2022). 35179911 10.1103/PhysRevLett.128.053901

[3] M. M. Tehrani, L. Mandel, Mode competition in a ring laser at line center. Opt. Lett. 1, 196–198 (1977). 19680375 10.1364/ol.1.000196

[4] M. Sorel, P. J. R. Laybourn, G. Giuliani, S. Donati, Unidirectional bistability in semiconductor waveguide ring lasers. Appl. Phys. Lett. 80, 3051–3053 (2002).

[5] G. Van der Sande, L. Gelens, P. Tassin, A. Scirè, J. Danckaert, Two-dimensional phase-space analysis and bifurcation study of the dynamical behaviour of a semiconductor ring laser. J. Phys. B: At. Mol. Opt. Phys. 41, 095402 (2008).

[6] S. V. Zhukovsky, D. N. Chigrin, J. Kroha, Bistability and mode interaction in microlasers. *Phys. Rev.* A 79, 033803 (2009).

[7] Q.-T. Cao, H. Wang, C.-H. Dong, H. Jing, R.-S. Liu, X. Chen, L. Ge, Q. Gong, Y.-F. Xiao, Experimental demonstration of spontaneous chirality in a nonlinear microresonator. Phys. Rev. Lett. 118, 033901 (2017). 28157372 10.1103/PhysRevLett.118.033901

[8] L. Del Bino, J. M. Silver, S. L. Stebbings, P. Del’Haye, Symmetry breaking of counter-propagating light in a nonlinear resonator. Sci. Rep. 7, 43142 (2017). 28220865 10.1038/srep43142 PMC5318886

[9] R. C. Keitel, B. le Feber, K. M. Dettlaff, R. Brechbühler, E. De Leo, H. Rojo, D. J. Norris, Single-pulse measurement of orbital angular momentum generated by microring lasers. ACS Nano 15, 19185–19193 (2021). 34780165 10.1021/acsnano.1c03792

[10] E. Wertz, L. Ferrier, D. D. Solnyshkov, R. Johne, D. Sanvitto, A. Lemaître, I. Sagnes, R. Grousson, A. V. Kavokin, P. Senellart, G. Malpuech, J. Bloch, Spontaneous formation and optical manipulation of extended polariton condensates. Nat. Phys. 6, 860–864 (2010).

[11] H. Ohadi, E. Kammann, T. C. H. Liew, K. G. Lagoudakis, A. V. Kavokin, P. G. Lagoudakis, Spontaneous symmetry breaking in a polariton and photon laser. Phys. Rev. Lett. 109, 016404 (2012). 23031120 10.1103/PhysRevLett.109.016404

[12] V. G. Sala, F. Marsault, M. Wouters, E. Galopin, I. Sagnes, A. Lemaître, J. Bloch, A. Amo, Stochastic precession of the polarization in a polariton laser. Phys. Rev. B 93, 115313 (2016).

[13] H. Sigurdsson, Y. S. Krivosenko, I. V. Iorsh, I. A. Shelykh, A. V. Nalitov, Spontaneous topological transitions in a honeycomb lattice of exciton-polariton condensates due to spin bifurcations. Phys. Rev. B 100, 235444 (2019).

[14] M. Haelterman, All-optical set-reset flip-flop operation in the nonlinear Fabry-Pérot interferometer. Opt. Commun. 86, 189–191 (1991).

[15] B. Maes, M. Soljačić, J. D. Joannopoulos, P. Bienstman, R. Baets, S.-P. Gorza, M. Haelterman, Switching through symmetry breaking in coupled nonlinear micro-cavities. Opt. Express 14, 10678–10683 (2006). 19529474 10.1364/oe.14.010678

[16] J. Stehr, J. Crewett, F. Schindler, R. Sperling, G. von Plessen, U. Lemmer, J. M. Lupton, T. A. Klar, J. Feldmann, A. W. Holleitner, M. Forster, U. Scherf, A low threshold polymer laser based on metallic nanoparticle gratings. Adv. Mater. 15, 1726–1729 (2003).

[17] J. Y. Suh, C. H. Kim, W. Zhou, M. D. Huntington, D. T. Co, M. R. Wasielewski, T. W. Odom, Plasmonic bowtie nanolaser arrays. Nano Lett. 12, 5769–5774 (2012). 23013283 10.1021/nl303086r

[18] W. Wang, N. Watkins, A. Yang, R. D. Schaller, G. C. Schatz, T. W. Odom, Ultrafast dynamics of lattice plasmon lasers. J. Phys. Chem. Lett. 10, 3301–3306 (2019). 31181939 10.1021/acs.jpclett.9b01076

[19] T. B. Hoang, G. M. Akselrod, A. Yang, T. W. Odom, M. H. Mikkelsen, Millimeter-scale spatial coherence from a plasmon laser. Nano Lett. 17, 6690–6695 (2017). 28956442 10.1021/acs.nanolett.7b02677

[20] J. M. Winkler, M. J. Ruckriegel, H. Rojo, R. C. Keitel, E. De Leo, F. T. Rabouw, D. J. Norris, Dual-wavelength lasing in quantum-dot plasmonic lattice lasers. ACS Nano 14, 5223–5232 (2020). 32159334 10.1021/acsnano.9b09698

[21] T. K. Hakala, H. T. Rekola, A. I. Väkeväinen, J.-P. Martikainen, M. Nečada, A. J. Moilanen, P. Törmä, Lasing in dark and bright modes of a finite-sized plasmonic lattice. Nat. Commun. 8, 13687 (2017). 28045047 10.1038/ncomms13687 PMC5216126

[22] R. Guo, M. Nečada, T. Hakala, A. Väkeväinen, P. Törmä, Lasing at K points of a honeycomb plasmonic lattice. Phys. Rev. Lett. 122, 013901 (2019). 31012715 10.1103/PhysRevLett.122.013901

[23] R. Heilmann, G. Salerno, J. Cuerda, T. K. Hakala, P. Törmä, Quasi-BIC mode lasing in a quadrumer plasmonic lattice. ACS Photon. 9, 224–232 (2022). 10.1021/acsphotonics.1c01416 PMC8780794 35083367

[24] A. H. Schokker, A. F. Koenderink, Lasing at the band edges of plasmonic lattices. Phys. Rev. B 90, 155452 (2014).

[25] A. H. Schokker, A. F. Koenderink, Statistics of randomized plasmonic lattice lasers. ACS Photon. 2, 1289–1297 (2015).

[26] A. H. Schokker, F. van Riggelen, Y. Hadad, A. Alù, A. F. Koenderink, Systematic study of the hybrid plasmonic-photonic band structure underlying lasing action of diffractive plasmon particle lattices. Phys. Rev. B 95, 085409 (2017).

[27] K. Guo, A. F. Koenderink, Spatial intensity distribution in plasmonic particle array lasers. Phys. Rev. Appl. 11, 024025 (2019).

[28] H. Kogelnik, C. V. Shank, Stimulated emission in a periodic structure. Appl. Phys. Lett. 18, 152–154 (1971).

[29] A. Overvig, A. Alù, Diffractive nonlocal metasurfaces. Laser Photon. Rev. 16, 2100633 (2022).

[30] A. Abass, S. R. K. Rodriguez, J. Gómez Rivas, B. Maes, Tailoring dispersion and eigenfield profiles of plasmonic surface lattice resonances. ACS Photon. 1, 61–68 (2014).

[31] G. Weick, C. Woollacott, W. L. Barnes, O. Hess, E. Mariani, Dirac-like plasmons in honeycomb lattices of metallic nanoparticles. Phys. Rev. Lett. 110, 106801 (2013). 23521276 10.1103/PhysRevLett.110.106801

[32] H. Saito, D. Yoshimoto, Y. Moritake, T. Matsukata, N. Yamamoto, T. Sannomiya, Valley-polarized plasmonic edge mode visualized in the near-infrared spectral range. Nano Lett. 21, 6556–6562 (2021). 34314178 10.1021/acs.nanolett.1c01841

[33] Q. T. Trinh, S. K. Nguyen, D. H. Nguyen, G. K. Tran, V. H. Le, H.-S. Nguyen, Q. Le-Van, Coexistence of surface lattice resonances and bound states in the continuum in a plasmonic lattice. Opt. Lett. 47, 1510–1513 (2022). 35290351 10.1364/OL.447933

[34] R. Kolkowski, S. Kovaios, A. F. Koenderink, Pseudochirality at exceptional rings of optical metasurfaces. Phys. Rev. Res. 3, 023185 (2021).

[35] L. Lu, J. D. Joannopoulos, M. Soljačić, Topological photonics. Nat. Photon. 8, 821–829 (2014).

[36] M.-A. Miri, A. Alù, Exceptional points in optics and photonics. Science 363, eaar7709 (2019). 30606818 10.1126/science.aar7709

[37] B. Bahari, A. Ndao, F. Vallini, A. E. Amili, Y. Fainman, B. Kanté, Nonreciprocal lasing in topological cavities of arbitrary geometries. Science 358, 636–640 (2017). 29025992 10.1126/science.aao4551

[38] P. St-Jean, V. Goblot, E. Galopin, A. Lemaître, T. Ozawa, L. Le Gratiet, I. Sagnes, J. Bloch, A. Amo, Lasing in topological edge states of a one-dimensional lattice. Nat. Photon. 11, 651–656 (2017).

[39] J.-S. Wu, V. Apalkov, M. I. Stockman, Topological Spaser. Phys. Rev. Lett. 124, 017701 (2020). 31976714 10.1103/PhysRevLett.124.017701

[40] M. Notomi, H. Suzuki, T. Tamamura, Directional lasing oscillation of two-dimensional organic photonic crystal lasers at several photonic band gaps. Appl. Phys. Lett. 78, 1325–1327 (2001).

[41] P. Gartner, Spontaneous symmetry breaking in the laser transition. Phys. Rev. B 99, 115313 (2019).

[42] K. Sakoda, “Symmetry of eigenmodes” in *Optical Properties of Photonic Crystals* (Springer, ed. 2, 2005), pp. 43–80.

[43] T. Ochiai, K. Sakoda, Dispersion relation and optical transmittance of a hexagonal photonic crystal slab. Phys. Rev. B 63, 125107 (2001).

[44] R. Graham, H. Haken, Laserlight—First example of a second-order phase transition far away from thermal equilibrium. Z. Physik. 237, 31–46 (1970).

[45] A. Vaskin, R. Kolkowski, A. F. Koenderink, I. Staude, Light-emitting metasurfaces. Nanophotonics 8, 1151–1198 (2019).

[46] Y. Zhang, A. Chen, W. Liu, C. W. Hsu, B. Wang, F. Guan, X. Liu, L. Shi, L. Lu, J. Zi, Observation of polarization vortices in momentum space. Phys. Rev. Lett. 120, 186103 (2018). 29775334 10.1103/PhysRevLett.120.186103

[47] M. Imada, A. Chutinan, S. Noda, M. Mochizuki, Multidirectionally distributed feedback photonic crystal lasers. Phys. Rev. B 65, 195306 (2002).

[48] G. Heliotis, R. Xia, G. A. Turnbull, P. Andrew, W. L. Barnes, I. D. W. Samuel, D. D. C. Bradley, Emission characteristics and performance comparison of polyfluorene lasers with one- and two-dimensional distributed feedback. Adv. Funct. Mater. 14, 91–97 (2004).

[49] E. Miyai, K. Sakai, T. Okano, W. Kunishi, D. Ohnishi, S. Noda, Lasers producing tailored beams. Nature 441, 946 (2006). 16791186 10.1038/441946a

[50] S. T. Ha, Y. H. Fu, N. K. Emani, Z. Pan, R. M. Bakker, R. Paniagua-Domíınguez, A. I. Kuznetsov, Directional lasing in resonant semiconductor nanoantenna arrays. Nat. Nanotechnol. 13, 1042–1047 (2018). 30127475 10.1038/s41565-018-0245-5

[51] J. F. Wheeldon, T. Hall, H. Schriemer, Symmetry constraints and the existence of Bloch mode vortices in linear photonic crystals. Opt. Express 15, 3531–3542 (2007). 19532596 10.1364/oe.15.003531

[52] D. Malterre, B. Kierren, Y. Fagot-Revurat, C. Didiot, F. J. García de Abajo, F. Schiller, J. Cordón, J. E. Ortega, Symmetry breaking and gap opening in two-dimensional hexagonal lattices. New J. Phys. 13, 013026 (2011).

[53] M. Proctor, M. Blanco de Paz, D. Bercioux, A. García-Etxarri, P. Arroyo Huidobro, Higher-order topology in plasmonic Kagome lattices. Appl. Phys. Lett. 118, 091105 (2021).

[54] X. G. Juarez, R. Li, J. Guan, T. Reese, R. D. Schaller, T. W. Odom, M-point lasing in hexagonal and honeycomb plasmonic lattices. ACS Photon. 9, 52–58 (2022).

[55] H. Kogelnik, C. V. Shank, Coupled-wave theory of distributed feedback lasers. J. Appl. Phys. 43, 2327–2335 (1972).

[56] A. Kodigala, T. Lepetit, Q. Gu, B. Bahari, Y. Fainman, B. Kanté, Lasing action from photonic bound states in continuum. Nature 541, 196–199 (2017). 28079064 10.1038/nature20799

[57] T. K. Hakala, A. J. Moilanen, A. I. Väkeväinen, R. Guo, J.-P. Martikainen, K. S. Daskalakis, H. T. Rekola, A. Julku, P. Törmä, Bose–Einstein condensation in a plasmonic lattice. Nat. Phys. 14, 739–744 (2018).

[58] L. Hill, G.-L. Oppo, P. Del’Haye, Multi-stage spontaneous symmetry breaking of light in Kerr ring resonators. Commun. Phys. 6, 208 (2023).

[59] A. Berkhout, A. F. Koenderink, Perfect absorption and phase singularities in plasmon antenna array etalons. ACS Photon. 6, 2917–2925 (2019).

