# Supplementary material for: Spontaneous symmetry breaking in plasmon lattice lasers
Source: Sci Adv. 2024 Jul 5;10(27):eadn2723. doi: 10.1126/sciadv.adn2723 (PMC11225787; doi:10.1126/sciadv.adn2723)
Supplement: Supplementary file 1 — Sections S1 to S8 Figs. S1 to S10 Table S1 [file sciadv.adn2723_sm.pdf]

Science Advances
AAAS

Supplementary Materials for

Spontaneous symmetry breaking in plasmon lattice lasers

Nelson de Gaay Fortman *et al.*

Corresponding author: A. Femius Koenderink, f.koenderink@amolf.nl

Sci. Adv. **10**, eadn2723 (2024)
DOI: 10.1126/sciadv.adn2723

This PDF file includes:

Sections S1 to S8
Figs. S1 to S10
Table S1

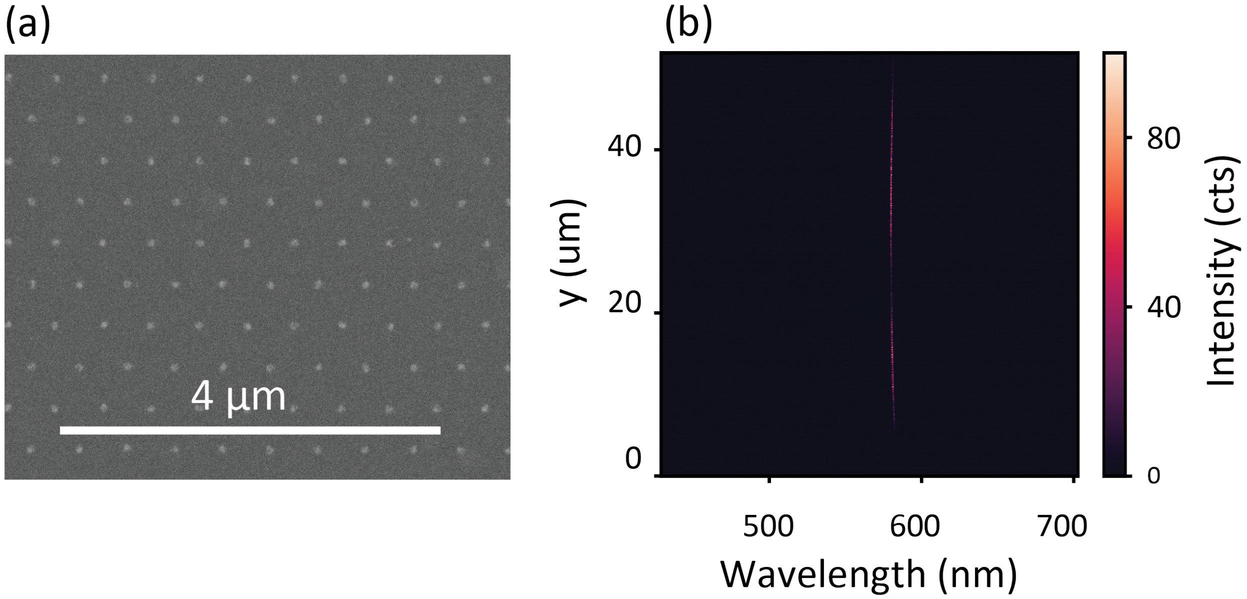

Supplementary figure S1. **Electron microscope image and spectrum of the lasing mode.** (a) SEM image of the K-point plasmonic lattice laser, showing the lattice of pitch 500 nm of silver plasmon particles with diameter 80 nm. (b) raw spectral image of K-point lasing emission, showing a single lasing line along the full length of the real-space field of view. The slight curvature is due to the spectrometer imaging optics and has not been corrected for in this image.

I. SPECTRUM OF THE LASING MODE

The K and K' modes both form a set of three k-space points that are coupled by a reciprocal lattice vector. There exist no lattice vectors that could couple K into K' and vice versa, which ensures the energy degeneracy between modes that live at the K/K'-points. In essence, the energy degeneracy is thus a result of Bloch's theorem. Fig S1(b) further evidences this degeneracy. While Fig 2(f) in the main manuscript shows the spectra taken in Fourier space, separating out K and K', Fig S1(b) shows spatially resolved spectroscopy, obtained by projecting a slice of the real space image onto the spectrometer slit. The spectral image is not corrected for imaging artifacts in the Andor spectrometer, and therefore there is a slight curvature in the result. Along the entire length of the field of view that is imaged (circa 44 μm) there is only a single lasing line.

What is more, we wish to explain the origin of the intensity imbalance between the spectra of K and K', seen in Fig 2(f) in the main text. The imbalance has no relation to

a global preference of the laser for a particular mode but is due to a trivial experimental reason: slicing k-space with a narrow spectrometer slit can lead to an apparent imbalance, as can averaging over just a few shots. That there is no imbalance on average, and no global preference, is evident from the statistical analysis in Fig 4: these data are extracted from full 2D Fourier imaging without any spectrometer slit to slice the data, and hence do not suffer from the artifact.

II. SSB POWER DEPENDENCE

Spontaneous symmetry breaking generally occurs when one input parameter to a physical system is continuously changed, and crossing some threshold value, the system's energy mode reduces in symmetry. Fig S2(a) displays such threshold behavior for the K/K' mode laser, mapped as function of pump power. We use the parameter θ defined in the main text. Below lasing threshold, fluorescence at all K-points is equally bright ($\theta = 0$). As the pump power exceeds the lasing threshold power of 1.0 mJ/cm^2, a continuous spread becomes visible in the SSB parameter θ that expresses the relative intensity between the K and K' mode (y-axis). The spread remains uniform over the entire θ range when increasing pump power. Fig S2(a) is measured with the 250 fs pump pulses also used for the main manuscript. For Fig S2(b), we place the same sample in a similar fluorescence Fourier microscope but with 532 nm pump pulses of 500 ps pulse length, and we apply the same linear basis projection method to the Fourier images. For this much longer pump pulse regime, the system also shows no bifurcation, and a wide range of θ values is sampled. The spread in θ is less than in Fig S2(a). We attribute this to a higher fluorescence intensity background when using 500 ps long pulses than 250 fs, which reduces K/K' contrast. This is supported by Fig S2(c) that shows the histogram for 0.4 mJ/cm^2, but with fluorescence background subtracted prior to extracting θ. This recovers a more uniform distribution of θ over the full range.

III. COUPLED-MODE MODEL

From scalar coupled-mode theory, the A_1 K/K'-point Bloch mode is described by three coupled waves (see Fig S3(a)):

$$E_K(\mathbf{r}) = e^{iK_\mathbf{1}\cdot\mathbf{r}} + e^{iK_\mathbf{2}\cdot\mathbf{r}} + e^{iK_\mathbf{3}\cdot\mathbf{r}}. \tag{1}$$

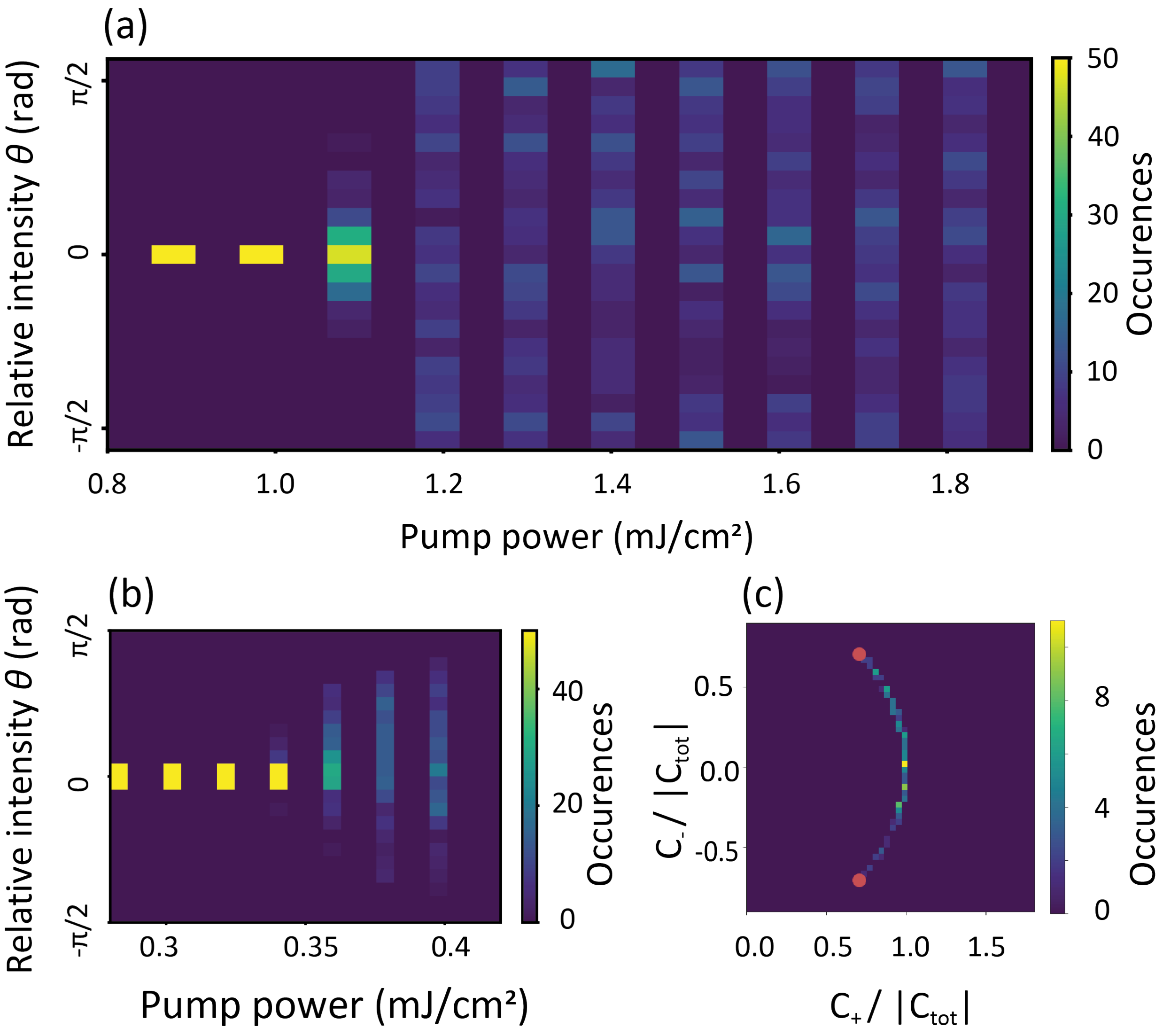

Supplementary figure S2. **Influence of pump strength on θ, for fs and ps pulse lengths.** (a) Histograms of K/K' intensity contrast parameter θ for increasing pump fluence, showing even spreads in θ for fluences well above lasing threshold. Data taken with the 250 fs pulsed laser setup described in the main text. (b) Same measurement as in (a) but taken with a setup using a laser of 500 ps pulse length. (c) Fluorescence intensity background subtracted occurrence histogram for θ, showing that the fluorescence background significantly reduces the spread in θ.

This simple model is used to calculate local field distributions for the A_1 mode. Figs S3(b,c) display the calculated intensity distributions of the K/K' modes, showing that they both form hexagonal patterns with peaks at the lattice nodes. The K/K' modes are thus spatially degenerate and are only distinguished by their opposite wavevector content. When

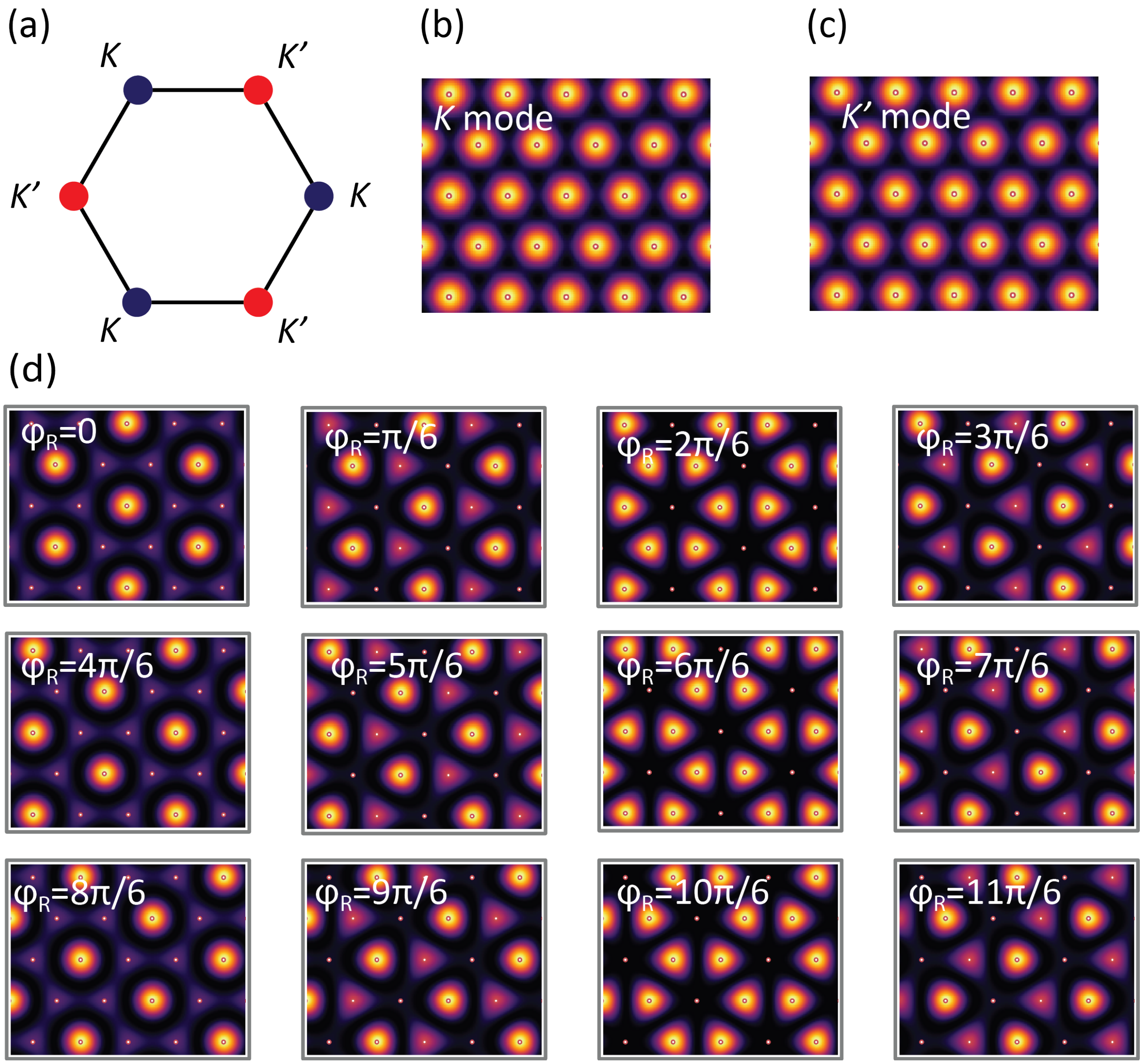

Supplementary figure S3. **Catalogue of K/K' mode interference patterns in real space.** (a) The hexagonal Brillouin zone corners comprise two sets of three reciprocal lattice vector-coupled K-points. The A-type modes of the K/K' points are calculated with a coupled-wave model, they have equal intensity distributions in space, as shown in (b) (K mode) and (c) (K' mode). (d) Catalogue of local $|E_T(\mathbf{r})|^2$ for different relative phases φ_R between K and K' modes (calculated with equal K-mode amplitudes $a = b$).

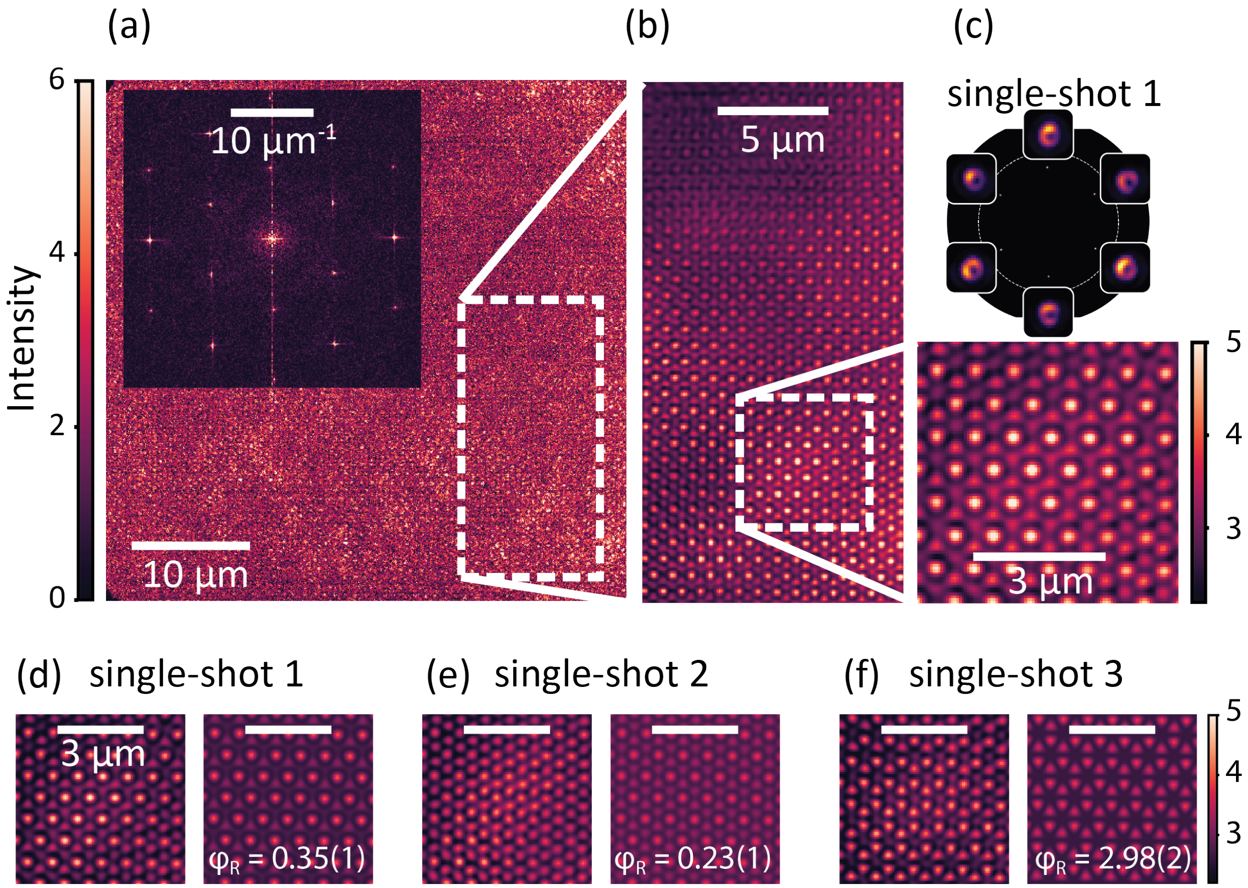

Supplementary figure S4. **Workflow illustration of the method to extract relative phase between the K and K' mode from the real space data.** (a) Raw image and FFT as inset. (b) Fourier filtered data for region as indicated in (a). (c) Close-up of area used for fitting, taken from (b). Also shown is the corresponding measured Fourier image. (d-f) show example data and fits.

both modes form a coherent superposition

$$E_T(\mathbf{r}) = aE_K(\mathbf{r}) + bE_{K'}(\mathbf{r})e^{i\varphi_R}, \tag{2}$$

the result in local intensity shows honeycomb interference patterns as visible in Fig S3(d). The supercell pattern has $\sqrt{3}$ times larger pitch than the sublattice, and the structure and spatial alignment relative to the origin of the patterns vary with relative phase φ_R.

IV. REAL SPACE FILTERING AND PHASE FITTING

In a single-shot real space image (Fig S4(a)), different periodic features are distinguishable, but their detailed structures are partially obscured by residual noise from scattering by fabrication disorder. We Fourier filter the real space data for fitting, as follows. The inset of Fig S4(a) shows the absolute value of the 2D Fourier transform F of the real space image, in which we clearly observe 19 sharp peaks (one at 0 wave vector, and the remaining 18 forming different orders spanning the honeycomb patterns across the hexagonal lattice). In the 2D FFT peaks, 6 points at $|\mathbf{k}_{//}| = 15\ \mu\text{m}^{-1}$ are the first order reciprocal lattice points of the real space lattice itself, and the remaining 12 are responsible for the superlattice periodicity. For fitting, we first define the size $l = 5.86\ \mu$m of the real space blocks of data that we will fit the coupled-mode model to. This defines a Fourier filter width $dk = 2\pi/l\ \mu\text{m}^{-1}$. We then remove high-frequency noise from the full real space image by selecting only regions of width Δk around each of the 19 sharp peaks (Gaussian filters, width dk). Inverse-Fourier transforming the masked FFT returns the filtered real space image. Intensities in real-space images in Fig S4 are reported in dimensionless units. For a quantitative scale, the data in Fig S4(a) corresponds to $1.4 \cdot 10^6$ camera ADU units integrated over the 44 by 44 μm^2 field of view. With a camera quantum efficiency of 70% this translates into circa $1 \cdot 10^6$ collected photons per laser shot (500 photons per square micron). The Fourier filtering suppresses the Poisson counting noise.

We then fit filtered real space intensity patterns of size $l \times l$ to the coupled-mode model with the Python SciPy minimize function using the BFGS algorithm. For all the fits, we choose the same area in the sample, and the block origin is chosen to coincide with an intensity peak in the ensemble-averaged data. This ensures a precise and common registration to the underlying particle lattice, which is needed to avoid ambiguity in fitting the phase. The fit function reads:

$$|E_T(\mathbf{r})|^2 = A|(\sqrt{a}E_K(\mathbf{r}) + \sqrt{b}E_{K'}(\mathbf{r})e^{i\varphi_R})|^2 + B, \tag{3}$$

where the relative amplitude coefficients a and b are derived from θ, taken from the Fourier-space measurement, as: $a = (1 + \tan\theta/2)/2$ and $b = (1 - \tan\theta/2)/2$. a and b are square rooted to obtain the right units. The only free fit parameters are the overall amplitude and background counts (A and B) and the relative phase φ_R. Representative fit results are

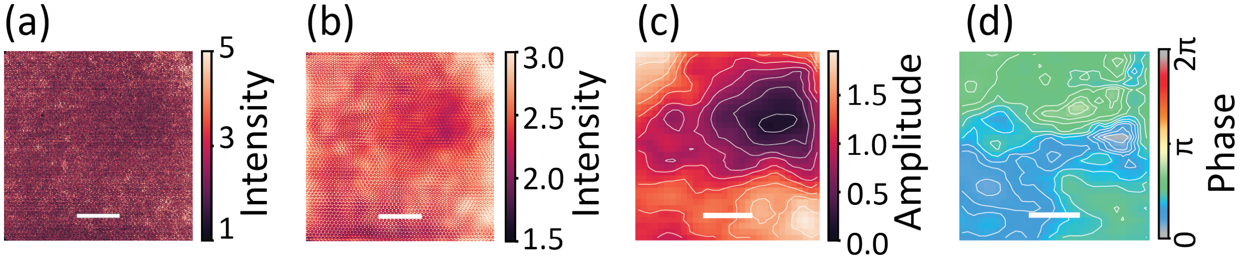

Supplementary figure S5. **Spatial amplitude and phase landscapes of a single lasing shot.** (a) The raw single-shot real space data, to which the spatial filter technique is applied to obtain (b). (c) Fitted amplitude of the periodic patterns above the fluorescence background. (d) Spatial landscape of the extracted relative phase between K and K' mode. Scale bars are 10 µm.

shown in Figs S4(d,e,f). We note that performing this fit in real space works best at choices of l that encompass of order 10×10 unit cells.

V. SPATIAL LANDSCAPES OF THE PHASE

The fitting procedure as described above can be extended to obtain a spatial phase texture over the microscope field of view. To this end, we divide the field of view into a target set of phase sampling points and for each sampling point we determine the phase by real space fitting the intensity pattern inside a small box centered at the sampling point (side length 5× larger than the sampling point distance). For successful fitting, it is important that in all images the underlying particle lattice is precisely referred to one common origin, since otherwise an error in spatial position would translate as a phase slip (see Fig S3). We have found it advantageous to first fit the phase texture at a coarse sampling resolution, fitting larger areas at a time, and using those fit results as an initial guess for fitting at refined resolutions. Generally, we find that the local phase can be fitted accurately except in two cases. First, whenever the lasing is purely K or K', the relative phase is φ_R not defined. In practice, this means that also close to pure K/K'-lasing the phase is hard to determine. Second, the amplitude maps show variations in laser intensity, including marked minima. We note that such inhomogeneities in amplitude envelopes are intrinsic to DFB lasing, and occur, for instance, in coupled mode theory descriptions from a nonlinear interplay of laser size, gain coefficient and feedback strength in coupled mode theory. At intensity minima,

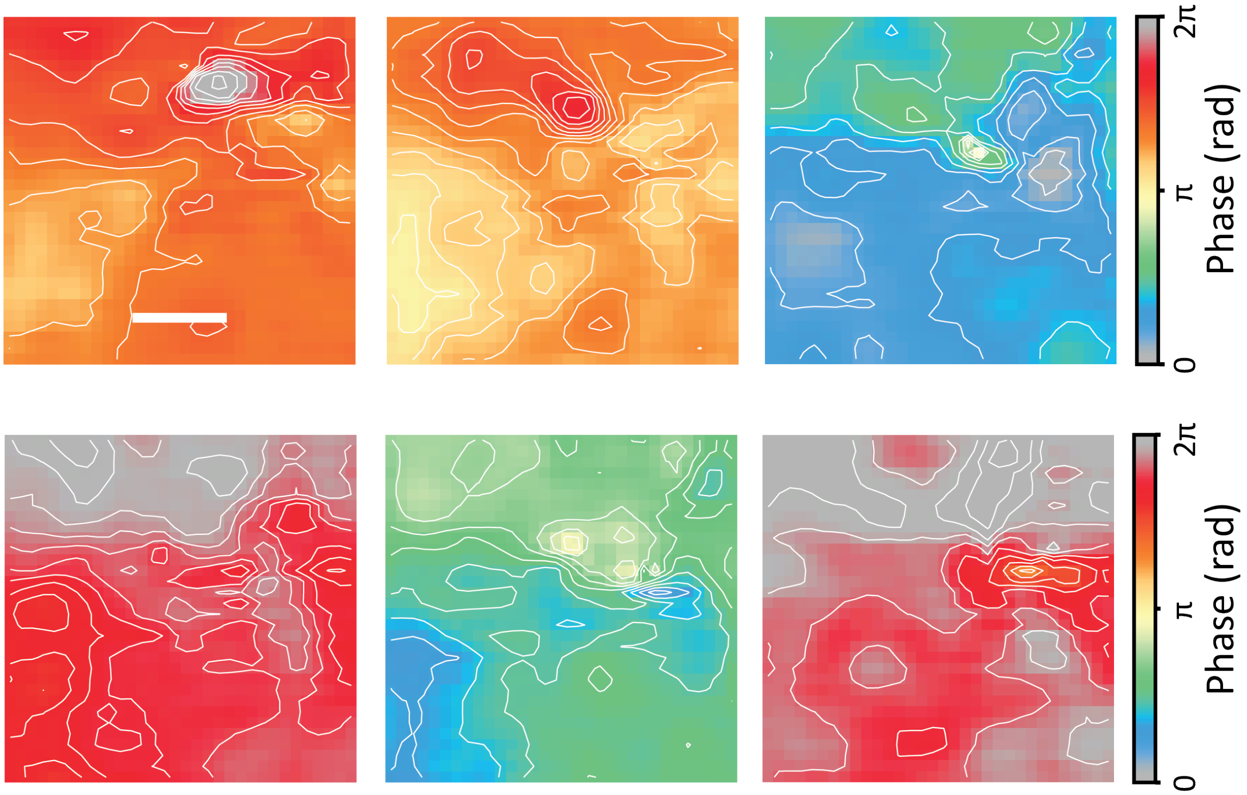

Supplementary figure S6. **Spatial phase landscapes for six subsequent single-shots.** These landscapes show random excursions from the mean spatial phase texture.

the contrast in the data to which the fit is performed is low. Fig S5 shows the results for one example frame, reporting the raw real space image, the Fourier filtered data, and the extracted phase map. For this particular example, there are phase excursions of order 0.1 to 0.2 radians around the mean. Fig S6 shows such phasemaps for 6 subsequent frames. Both the average phase, and the phase fluctuations vary from map to map. The spatial autocorrelation lengths are of order 5 μm.

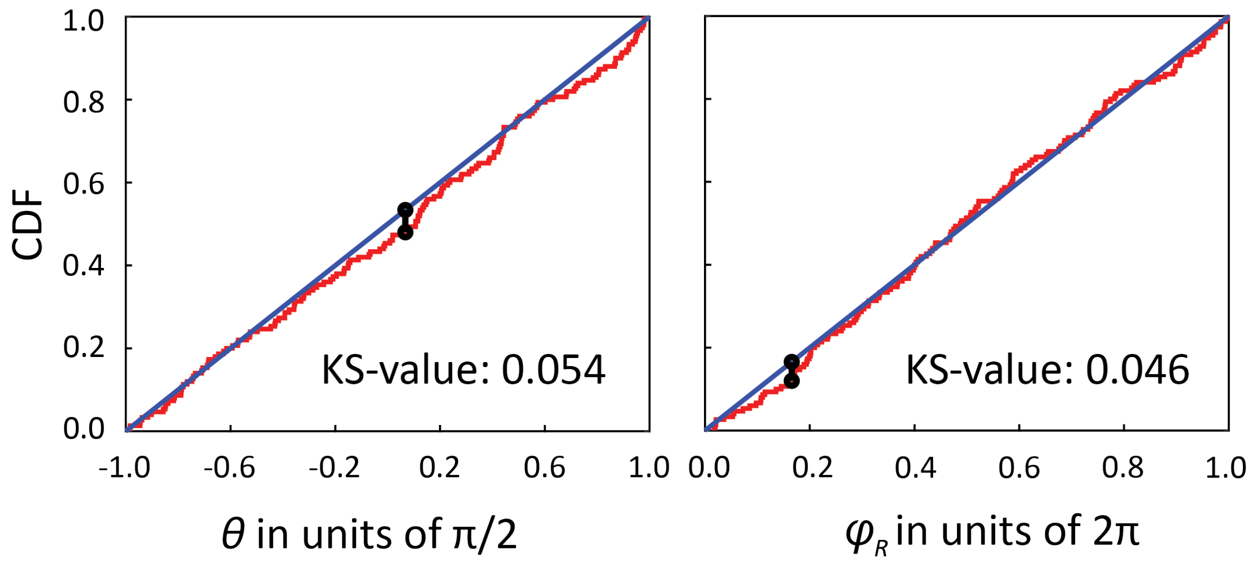

Supplementary figure S7. **Cumulative distribution functions for Kolmogorv-Smirnov testing.** The cumulative distribution functions of hypothetical perfectly uniform distribution datasets are shown in blue, and of the SSB data in red. The data is taken from the highest pump power dataset in Fig S2a. Both θ and φ_R pass the Kolmogorov-Smirnov test.

VI. STATISTICS OF SSB PARAMETERS (KOLMOGOROV-SMIRNOV TEST)

Our sampling of the probability distribution typically contains of order 1000 shots (Fig 4(b,c): 900 shots, combined from the six highest pump powers (each 150 shots) in Fig S2a). Focusing on the symmetry breaking in relative intensity and phase as gauged by θ and φ_R, one can perform statistical testing to test if the data is likely drawn from a uniform distribution. A standard test is the Kolmogorov-Smirnov test (KS), which compares measured and hypothesized cumulative probability distribution functions. As an example of such an analysis, in Fig S7 we plot the KS test applied to data taken from the highest power dataset in Fig S2a (150 shots). The measured cumulative distribution function matches that of a uniform distribution for θ and φ_R well, with a respective maximum deviations D = 0.054 and D = 0.046, which are smaller than the threshold value of the Kolmogorov-Smirnov test evaluated at $\alpha = 0.05$ (5%) as significance level: D = 0.11. Most runs pass the test at this level of strictness for θ and φ_R, and a few runs marginally fail the test. In our view this means that quantitatively the data is in good accord with the hypothesis of a uniform distribution. In a future study, it would be interesting to resolve if there is finer structure

in phase space, e.g., in relation to how the system geometrical symmetry is broken, or how much disorder is introduced.

VII. MINIMAL MODEL OF COUPLED RATE EQUATIONS

The dynamics of our system involve the competition between the K and K' modes and the spontaneous symmetry breaking under pulsed pumping. To create a minimal model accounting for these effects, we adapt the approach presented in Ref [39]. In that work, a system very similar to ours was considered, comprising a honeycomb lattice of plasmonic nanoshells with gain-medium cores, in which the K/K' mode competition gives rise to bistable lasing under continuous pumping. We adapt the model taking into account the following distinguishing features of our system:

1. The lattice is hexagonal (there is only one lattice site per unit cell).

2. The electric fields are a priori defined as an interference of the the K-point plane waves of the A_1 irreducible representation:

$$E(\mathbf{r}, t) = a(t)E_K(\mathbf{r}) + b'(t)E_{K'}(\mathbf{r}), \tag{4}$$

$$E_K(\mathbf{r}) = E_{K'}(\mathbf{r})^* = e^{iK_\mathbf{1}\cdot\mathbf{r}} + e^{iK_\mathbf{2}\cdot\mathbf{r}} + e^{iK_\mathbf{3}\cdot\mathbf{r}}, \tag{5}$$

where $b'(t) = b(t)e^{i\varphi_R}$. For simplicity, we neglect the vector nature of the electric field and treat it as a spatially varying complex-valued scalar field.

3. The gain medium is spatially extended beyond the plasmonic nanoparticles.

4. The pumping is pulsed, with pulse duration that may be comparable to or shorter than the population dynamics of the gain medium. Therefore, a time-dependent pumping rate $g(t)$ must be considered:

$$g(t) = g_{\max}e^{-(t-t_0)/\tau^2}, \tag{6}$$

where $g_{\max}$ is the peak pumping rate, t_0 is the time of the pulse peak arrival, and τ quatifies the pulse duration. For simplicity, we assume that the pumping rate is spatially uniform.

To obtain the coupled system of equations describing both the gain medium and the K and K' modes, we follow the density matrix formalism presented in Ref [39]. The gain medium is treated as a two-level system, with a ground state $|1\rangle$ and an excited state $|2\rangle$. Using the rotating wave approximation, the nondiagonal elements of the density matrix can be written as $[\rho(\mathbf{r}, t)]_{12} = \bar{\rho}(\mathbf{r}, t)e^{i\omega_K t}$, where $\omega_K = \omega_{K'}$ is the frequency of the K and K'

modes. Defining the population inversion as $n(\mathbf{r},t) = \rho_{22}(\mathbf{r},t) - \rho_{11}(\mathbf{r},t)$, the system of equations can be expressed as:

$$\frac{da(t)}{dt} = -\gamma_K a(t) + \frac{i}{\hbar} N_g \int \bar{\rho}(\mathbf{r},t)^* \mu_g E_K(\mathbf{r})^* d\mathbf{r}, \tag{7}$$

$$\frac{db'(t)}{dt} = -\gamma_K b'(t) + \frac{i}{\hbar} N_g \int \bar{\rho}(\mathbf{r},t)^* \mu_g E_{K'}(\mathbf{r})^* d\mathbf{r}, \tag{8}$$

$$\frac{dn(\mathbf{r},t)}{dt} = g(t)[1-n(\mathbf{r},t)] - \gamma_{nr}[1+n(\mathbf{r},t)] - \frac{1}{\hbar} 4\mathrm{Im}\{\bar{\rho}(\mathbf{r},t)\mu_g[a(t)E_K(\mathbf{r}) + b'(t)E_{K'}(\mathbf{r})]\}, \tag{9}$$

$$\frac{d\bar{\rho}(\mathbf{r},t)}{dt} = -\Gamma_{12}\bar{\rho}(\mathbf{r},t) + \frac{i}{\hbar} n(\mathbf{r},t)\mu_g[a(t)^* E_K(\mathbf{r})^* + b'(t)^* E_{K'}(\mathbf{r})^*], \tag{10}$$

where $\gamma_K = \gamma_{K'}$ is the loss rate of the K and K' modes, N_g is the number of gain medium molecules inside the integrated volume, μ_g is the linear transition dipole moment of the gain medium molecules (which is assumed to couple equally to the K and K' modes), γ_{nr} is the nonradiative decay rate of the excited state $|2\rangle$, and Γ_{12} is the polarization relaxation rate for the $|2\rangle \rightarrow |1\rangle$ transition.

For any combination of the K and K' modes, the electric field distribution is periodic with a supercell defined by the green contour in Fig S8. The spatial distributions of $E_K(\mathbf{r})$, $E_{K'}(\mathbf{r})$, $\bar{\rho}(\mathbf{r},t)$ and $n(\mathbf{r},t)$ must be considered only inside this supercell. The area of this supercell is three times larger than that of the original unit cell (dashed blue contour) of the underlying plasmonic lattice. In Fig S8, we show the lattice sites as the red circles located at the high-intensity spots.

We further simplify our model by approximating the spatial distributions of $E_K(\mathbf{r})$, $E_{K'}(\mathbf{r})$, $\bar{\rho}(\mathbf{r},t)$ and $n(\mathbf{r},t)$ by their corresponding values at the three lattice sites included in the supercell, with $\mathbf{r}_1 = -\frac{\Lambda}{2}(1,\sqrt{3})$, $\mathbf{r}_2 = (0,0)$, $\mathbf{r}_3 = \frac{\Lambda}{2}(1,\sqrt{3})$, where $\Lambda = 500$ nm is the period of the plasmonic nanoparticle lattice. This yields the following system of eight coupled equations:

$$\frac{da(t)}{dt} = -\gamma_K a(t) + \frac{i}{\hbar} N_g \sum_{m=1}^{3} \bar{\rho}(\mathbf{r},t)^* \mu_g E_K(\mathbf{r})^*, \tag{11}$$

$$\frac{db'(t)}{dt} = -\gamma_K b'(t) + \frac{i}{\hbar} N_g \sum_{m=1}^{3} \bar{\rho}(\mathbf{r},t)^* \mu_g E_{K'}(\mathbf{r})^*, \tag{12}$$

$$\frac{dn(\mathbf{r}_m,t)}{dt} = g(t)[1-n(\mathbf{r}_m,t)] - \gamma_{nr}[1+n(\mathbf{r}_m,t)] - \frac{1}{\hbar} 4\mathrm{Im}\{\bar{\rho}(\mathbf{r}_m,t)\mu_g[a(t)E_K(\mathbf{r}_m) + b'(t)E_{K'}(\mathbf{r}_m)]\}, \tag{13}$$

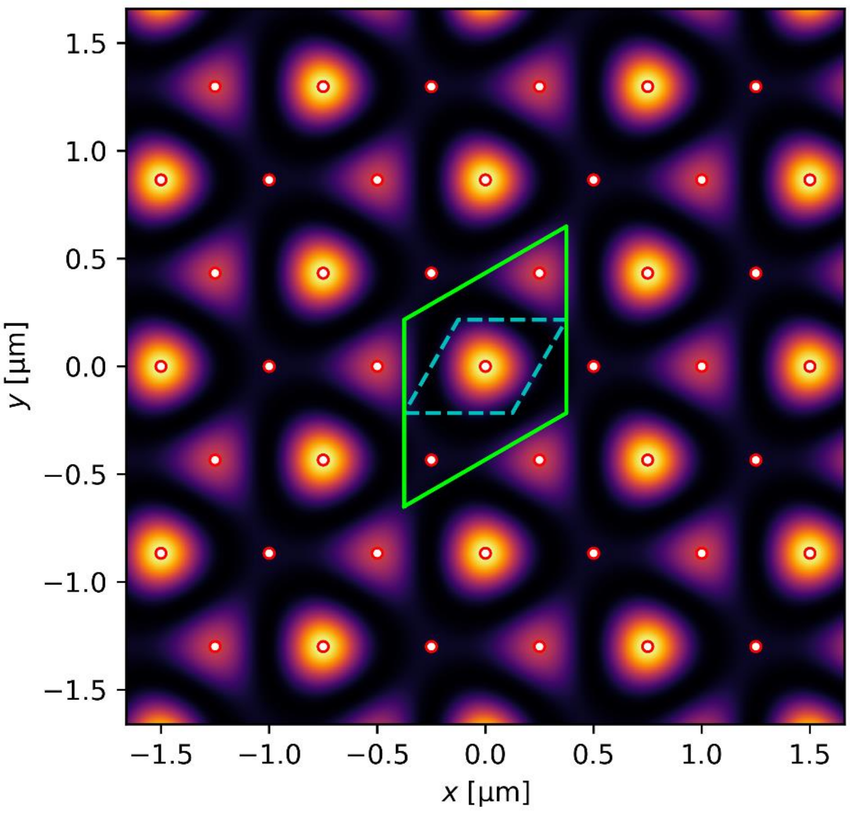

Supplementary figure S8. **Illustration of the supercell (green contour) that is considered in the minimal model.** The lattice nodes of the underlying plasmonic nanoparticle lattice (red circles) and its unit cell (dashed blue contour) are shown for comparison. The colormap shows the example intensity distribution for $a = b$ and $\varphi_R = \pi/6$

$$\frac{d\bar{\rho}(\mathbf{r}_m, t)}{dt} = -\Gamma_{12}\bar{\rho}(\mathbf{r}_m, t) + \frac{i}{\hbar} n(\mathbf{r}_m, t)\mu_g [a(t)^* E_K(\mathbf{r}_m)^* + b'(t)^* E_{K'}(\mathbf{r}_m)^*], \tag{14}$$

where $m \in \{1, 2, 3\}$. Note that $K_1 = -K_1' = \frac{4\pi}{3\Lambda}(1, 0)$, $K_2 = -K_2' = \frac{2\pi}{3\Lambda}(-1, \sqrt{3})$, $K_3 =$

$-K_3' = \frac{2\pi}{3\Lambda}(-1, -\sqrt{3})$, which allows us to express $E_K(\mathbf{r})$ and $E_{K'}(\mathbf{r})$ as:

$$E_K(\mathbf{r}_1) = e^{iK_1\mathbf{r}_1} + e^{iK_2\mathbf{r}_1} + e^{iK_3\mathbf{r}_1} = 3e^{-i\frac{2\pi}{3}}$$

$$E_K(\mathbf{r}_2) = e^{iK_1\mathbf{r}_2} + e^{iK_2\mathbf{r}_2} + e^{iK_3\mathbf{r}_2} = 3$$

$$E_K(\mathbf{r}_3) = e^{iK_1\mathbf{r}_3} + e^{iK_2\mathbf{r}_3} + e^{iK_3\mathbf{r}_3} = 3e^{i\frac{2\pi}{3}}$$

$$E_{K'}(\mathbf{r}_1) = e^{-iK_1\mathbf{r}_1} + e^{-iK_2\mathbf{r}_1} + e^{-iK_3\mathbf{r}_1} = 3e^{i\frac{2\pi}{3}}$$

$$E_{K'}(\mathbf{r}_2) = e^{-iK_1\mathbf{r}_2} + e^{-iK_2\mathbf{r}_2} + e^{-iK_3\mathbf{r}_2} = 3$$

$$E_{K'}(\mathbf{r}_3) = e^{-iK_1\mathbf{r}_3} + e^{-iK_2\mathbf{r}_3} + e^{-iK_3\mathbf{r}_3} = 3e^{-i\frac{2\pi}{3}}.$$

Since $|E_K(\mathbf{r})| = |E_{K'}(\mathbf{r})|$ we can define the Rabi frequency as:

$$\Omega = \frac{\mu_g |E_K(\mathbf{r})|}{\hbar}. \tag{15}$$

Abbreviating $n(\mathbf{r}_m, t)$ as $n_m(t)$ and $\bar{\rho}(\mathbf{r}_m, t)$ as $\bar{\rho}_m(t)$, the full system of equations can be written as:

$$\frac{da(t)}{dt} = -\gamma_K a(t) + \frac{i}{\hbar} N_g \Omega \Big[\bar{\rho}_1(t)^* e^{i\frac{2\pi}{3}} + \bar{\rho}_2(t)^* + \bar{\rho}_3(t) e^{-i\frac{2\pi}{3}}\Big], \tag{16}$$

$$\frac{db'(t)}{dt} = -\gamma_K b'(t) + \frac{i}{\hbar} N_g \Omega \Big[\bar{\rho}_1(t)^* e^{-i\frac{2\pi}{3}} + \bar{\rho}_2(t)^* + \bar{\rho}_3(t) e^{i\frac{2\pi}{3}}\Big], \tag{17}$$

$$\frac{dn_1(t)}{dt} = g(t)[1 - n_1(t)] - \gamma_{nr}[1 + n_1(t)] - 4\Omega \mathrm{Im}\Big\{\bar{\rho}_1(t)\Big[a(t)e^{-i\frac{2\pi}{3}} + b'(t)e^{i\frac{2\pi}{3}}\Big]\Big\}, \tag{18}$$

$$\frac{dn_2(t)}{dt} = g(t)[1 - n_2(t)] - \gamma_{nr}[1 + n_2(t)] - 4\Omega \mathrm{Im}\Big\{\bar{\rho}_2(t)\Big[a(t) + b'(t)\Big]\Big\}, \tag{19}$$

$$\frac{dn_3(t)}{dt} = g(t)[1 - n_3(t)] - \gamma_{nr}[1 + n_3(t)] - 4\Omega \mathrm{Im}\Big\{\bar{\rho}_3(t)\Big[a(t)e^{i\frac{2\pi}{3}} + b'(t)e^{-i\frac{2\pi}{3}}\Big]\Big\}, \tag{20}$$

$$\frac{d\bar{\rho}_1(t)}{dt} = -\Gamma_{12}\bar{\rho}_1(t) + in_1(t)\Omega\Big[a(t)^* e^{i\frac{2\pi}{3}} + b'(t)^* e^{-i\frac{2\pi}{3}}\Big], \tag{21}$$

$$\frac{d\bar{\rho}_2(t)}{dt} = -\Gamma_{12}\bar{\rho}_2(t) + in_2(t)\Omega\Big[a(t)^* + b'(t)^*\Big], \tag{22}$$

$$\frac{d\bar{\rho}_3(t)}{dt} = -\Gamma_{12}\bar{\rho}_3(t) + in_3(t)\Omega\Big[a(t)^* e^{-i\frac{2\pi}{3}} + b'(t)^* e^{i\frac{2\pi}{3}}\Big]. \tag{23}$$

We solve the coupled system of equations numerically in the complex domain in Python using the SciPy function scipy.integrate.solve_ivp, assuming the initial values $a(0) = b'(0) = 10^{-6}$ and $n_m(0) = \bar{\rho}_m(0) = 0$, for all m. To break the symmetry, we add noise terms $\delta\zeta_1 e^{i2\pi\zeta_2}$ and $\delta\zeta_3 e^{i2\pi\zeta_4}$ to the first and second equation, respectively, where $\delta = 10^{-8}$ is the noise amplitude and ζ_1 - ζ_4 are pseudorandom real numbers between 0 and 1 generated in each iteration of the solver. To end up in (θ, φ_R) space, we read the values $a(t_1 = 2.5\text{ ps})$

parameter	250 fs	500 ps	CW
τ	250 fs	500 ps	∞
t_0	2 ps	4 ns	0
t_1	2.5 ps	4.32 ns	15 ps
$g_{\max}$	1000 THz	150 THz	10 THz
γ_K	2 THz	1.35 THz	2 THz
γ_{nr}	5 THz	5 THz	5 THz
Γ_{12}	200 THz	200 THz	200 THz
N_g	10^6	10^6	10^6
Ω	0.05 THz	0.01 THz	0.05 THz
δ	10^{-8}	10^{-8}	10^{-8}

Supplementary table S1. **Values of the parameters used in the minimal model for three pumping schemes**. Under pulsed pumping, these parameters result in a uniform spread of θ and φ (reproducing the experimental observations) and under continuous wave pumping, a bifurcation is observed (typically reported for SSB in lasers). Here, t_1 is the time at which the values of θ and φ are probed.

and $b'(t_1 = 2.5$ ps), a timing when the system is fully in the lasing state. We project $w = (|a|^2, |b'|^2)$ onto the same linear basis to extract θ as described in Methods. In table S1 we list the values of the parameters that we used for the model.

Fig S9 shows two different examples of calculated $a(t)$ and $b'(t)$ obtained by solving the coupled rate equations with added random noise. Figs (a,c) show the absolute values of $a(t)$ and $b'(t)$, which determine the relative intensity SSB parameter θ, together with the pumping rate $g(t)$. Probing at a time $t_1 = 2.5$ ps (at which the system is clearly in the lasing regime), we find that $|a(t)|$ and $|b'(t)|$ take random values with respect to each other for each consecutive simulation. In the same example calculations, we find that the relative phase φ_R (in Figs (b,d)) seems to manifest randomly as well (also probed at $t_1 = 2.5$ ps). The aforementioned observations clearly hint towards similar random distributions in SSB phase space as observed in the experimental data with fs pump (main text, Fig 4). To compare the model to our experiment, we run a sequence of 900 separate calculations, extracting θ and

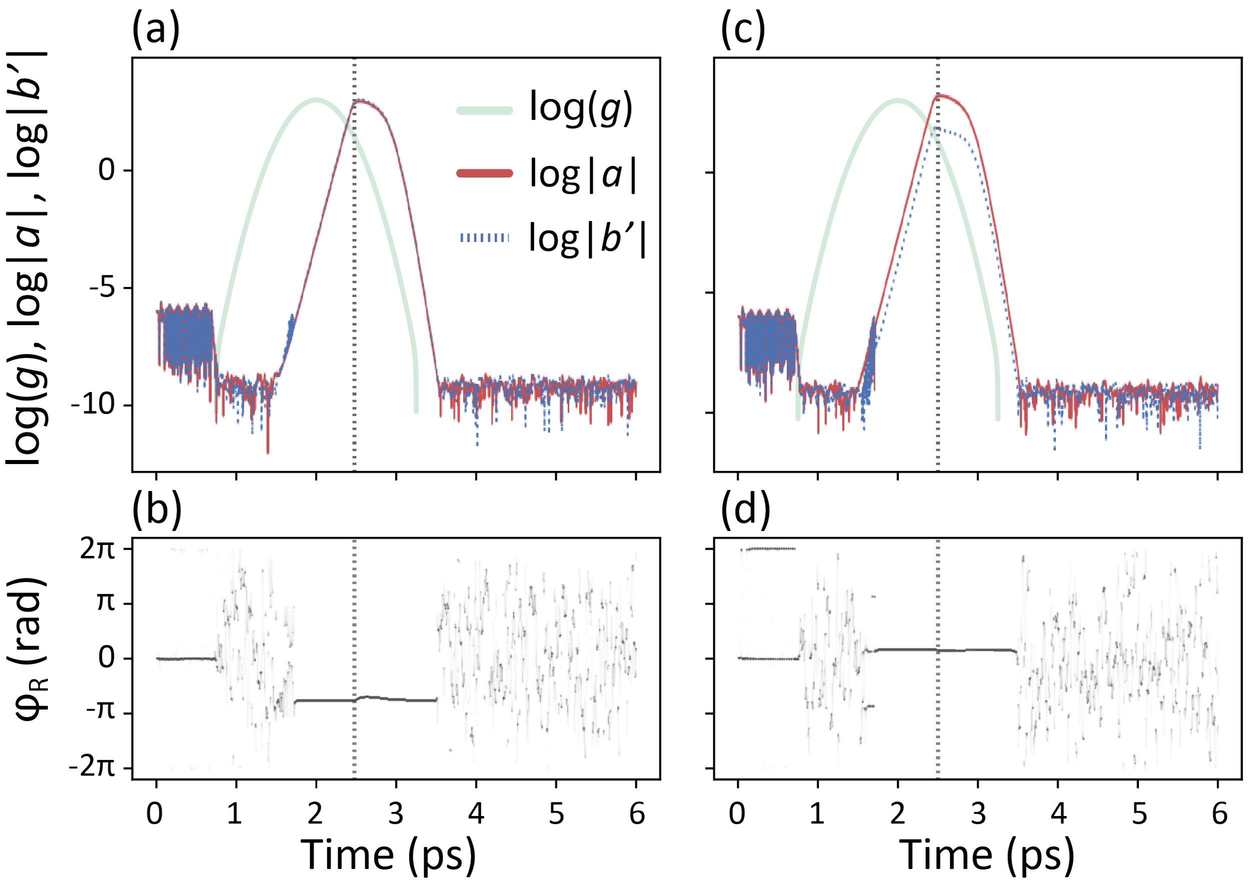

Supplementary figure S9. **Calculated time evolutions of mode parameters for fs pumping.** (a,c): two examples of the calculated time evolution of $|a(t)|$ and $|b'(t)|$ together with the pumping rate $g(t)$ (250 fs pulsed pump). (b,d): the relative phase φ_R between $|a(t)|$ and $|b'(t)|$. In all panels, a vertical line at $t_1 = 2.5$ ps is drawn; this is the time at which the calculated values are evaluated.

φ_R for each run at $t_1 = 2.5$ ps. In Fig S10, the datapoints from this sequence of calculations are plotted in time traces in (a,b) and on the SSB unit sphere in (c). Indeed, we observe a uniform spread in the SSB phase space unit sphere.

In SI section II, we have shown that pumping with 500 ps pulses leads to experimental observation of a similar uniform distribution over (θ, φ_R) phase space (Fig S2). Choosing the appropriate parameters (listed in table S1), the same behavior can be reproduced with our model, yielding an even spread over phase space similar to Fig S10. On the other hand, the same model also predicts that under continuous wave pumping, the system will eventually undergo the usual bifurcation, with one of the modes taking over all the energy from the gain medium. Such bifurcation can also be observed by choosing the pumping rate that

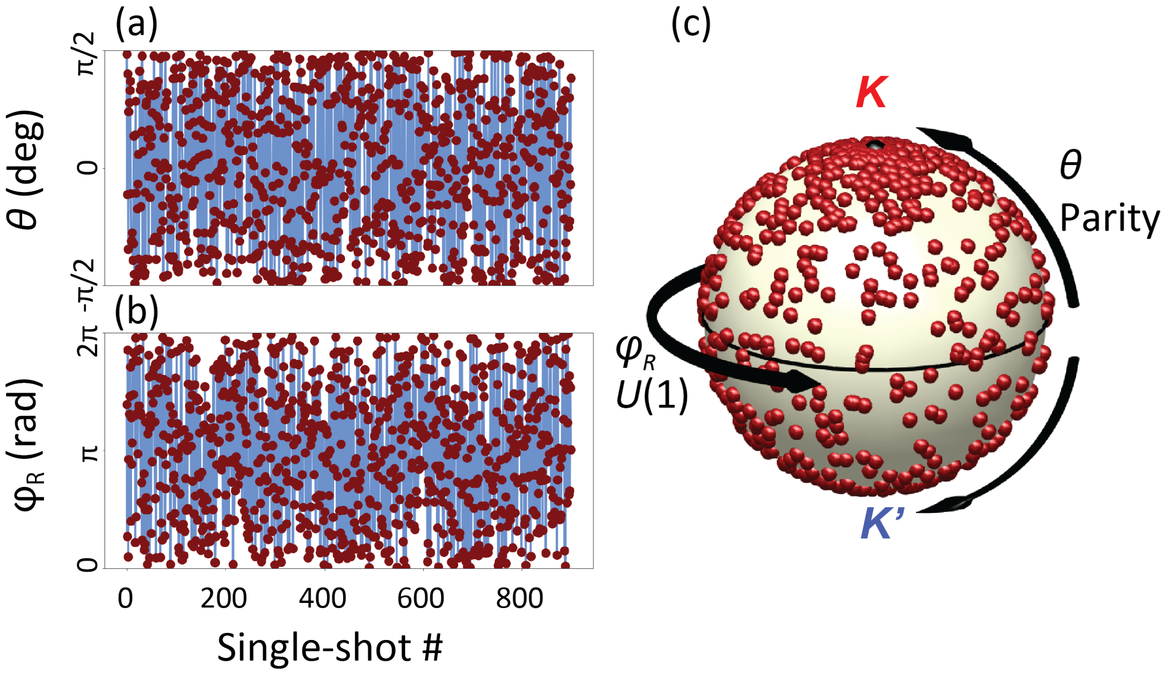

Supplementary figure S10. **Calculated SSB parameters and their distribution in phase space.** (a,b): a series of 900 calculations with the model, from which the SSB order parameters θ and φ_R are extracted. (c): same data mapped onto the surface of a unit sphere.

varies sufficiently slowly compared to other system dynamics.

We note that the presented model contains no geometrical disorder, yet reproduces our experiment. The symmetry breaking hence comes from the seed amplitude and phase noise (in experiment due to the random nature of the spontaneous emission that initiates the lasing). Amplitude noise alone (meaning: both modes independently seeded with amplitude noise, but fully correlated in phase, $\zeta_2 = \zeta_4$) is sufficient to cause SSB in θ. Obtaining SSB in φ_R requires the noise to be uncorrelated in phase. Many interesting physics aspects such as the precise spatially resolved physics of plasmon particles and SLRs, geometrical disorder, and the overall size and boundaries of the lasing structure cannot be incorporated in the model. This would require very large scale finite size FDTD simulations with gain and seed noise distributed over the spatial domain. This is far beyond the scope of this work. The density matrix model should be regarded as a minimal model. Yet on basis of the symmetry of the K and K' modes that it contains, the model already reproduces all the main physics of the experiment.

VIII. PYTHON CODE FOR THE MINIMAL MODEL

```
import matplotlib.pyplot as p

from numpy import exp,conj,pi,imag,log10,angle

import numpy as np

from scipy.integrate import solve_ivp as si

from numpy.random import random as rn

import seaborn as sns

from functools import cached_property

sns.set_theme()

sns.set(style="ticks", font="Calibri", context="talk",\

font_scale=1.5, rc={"lines.linewidth": 2})


class DensityMatrixApproximation:

    def __init__(self,t_,Ng,gK,Om,gnr,G12,t0,tau,gmax,noise_level,phiR0,a0,b0,tx=2.5):

        self.t_ = t_

        self.Ng = Ng

        self.gK = gK

        self.Om = Om

        self.gnr = gnr

        self.G12 = G12

        self.t0 = t0

        self.tau = tau

        self.gmax = gmax

        self.noise_level = noise_level

        self.phiR0 = phiR0

        self.a0 = a0

        self.b0 = b0

        self.tx = tx

        self.tx_i = np.where(t_==tx)[0][0]
```

```
def gt(self, t):
    return t*0+self.gmax*(abs(exp(-(t-self.t0)**2/self.tau**2)-exp(-25))+\
    (exp(-(t-self.t0)**2/self.tau**2)-exp(-25)))/2

def f(self, t, y):
    """
    Set of ordinary differential equations
    t: time in ps
    y: variables to solve: a(t),b(t),n1(t),n2(t),n3(t),rho1(t),rho2(t),rho3(t)
    """

    rn1 = rn()
    rn2 = rn()
    rn3 = rn()
    rn4 = rn()
    #rn3 = rn1
    #rn4 = rn2
    noise_a = self.noise_level*rn1*exp(2j*pi*rn2)
    noise_b = self.noise_level*rn3*exp(2j*pi*rn4)

    return [-self.gK*y[0]+noise_a+1j*self.Ng*self.Om*(conj(y[5])*exp(1j*2*pi/3)+\
    conj(y[6])+conj(y[7])*exp(-1j*2*pi/3)),\
    -self.gK*y[1]+noise_b+1j*self.Ng*self.Om*(conj(y[5])*exp(-1j*2*pi/3)+\
    conj(y[6])+conj(y[7])*exp(1j*2*pi/3)),\
    self.gt(t)*(1-y[2])-self.gnr*(1+y[2])-4*self.Om*imag(y[5]*\
    (y[0]*exp(-1j*2*pi/3)+y[1]*exp(1j*2*pi/3))),\
    self.gt(t)*(1-y[3])-self.gnr*(1+y[3])-4*self.Om*imag(y[6]*(y[0]+y[1])),\
    self.gt(t)*(1-y[4])-self.gnr*(1+y[4])-4*self.Om*imag(y[7]*\
    (y[0]*exp(1j*2*pi/3)+y[1]*exp(-1j*2*pi/3))),\
    -self.G12*y[5]+1j*y[2]*self.Om*(conj(y[0])*exp(1j*2*pi/3)+\
    conj(y[1])*exp(-1j*2*pi/3)),\
```

```
        -self.G12*y[6]+1j*y[3]*self.Om*(conj(y[0])+conj(y[1])),\
        -self.G12*y[7]+1j*y[4]*self.Om*(conj(y[0])*exp(-1j*2*pi/3)+\
        +conj(y[1])*exp(1j*2*pi/3))]

    @cached_property
    def solve_equations(self):
        r=si(self.f,(min(self.t_),max(self.t_)),\
        np.array([self.a0,self.b0,0.0+0.0j,0.0+0.0j,\
        0.0+0.0j,0.0+0.0j,0.0+0.0j,0.0+0.0j]),\
        method="RK45",t_eval=self.t_)
        return r

    def plot_time_evolution(self):
        r = self.solve_equations
        fig=p.figure(figsize=(10,10))
        plot1=p.subplot(211)
        ax0,=p.plot(r.t,log10(self.gt(r.t)),"g",alpha=0.25,lw=4)
        ax1,=p.plot(r.t,log10(abs(r.y[0])),"r",lw=2, rasterized="True")
        ax2,=p.plot(r.t,log10(abs(r.y[1])),":b",lw=2, rasterized="True")
        p.vlines(self.tx, -10, 4)
        p.legend([ax0,ax1,ax2],\
        ["log($g$)","log$|a|$","log$|b|$"],loc="upper right")
        p.ylabel("log($g$), log$|a|$, log$|b|$")
        p.subplot(212)
        p.scatter(r.t,angle(r.y[1])-angle(r.y[0]),\
        c="k",s=1,alpha=0.01, rasterized="True")
        p.yticks([-2*pi,-pi,0,pi,2*pi],["$-2\pi$","$-\pi$",0,"$\pi$","$2\pi$"])
        p.ylim(-2.2*pi,2.2*pi)
        p.ylabel("$\phi_R$",labelpad=-5)
        p.xlabel("$t$ [ps]")
        p.vlines(self.tx, -6.2, 6.2)
```

```
        p.subplots_adjust(left=0.2,right=0.95)
        p.savefig("time_evolution.pdf")

    def linear_projection(self, w):
        v0 = np.array(([1, 1]))
        v1 = np.array(([1,-1]))
        v0, v1 = v0/np.linalg.norm(v0),\
        v1/np.linalg.norm(v1)  # normalize v0 and v1
        c0 = np.tensordot(w, v0,\
        axes=([0],[0]))/np.dot(v0, v0)  # coefficient of projection w on v0
        c1 = np.tensordot(w, v1,\
        axes=([0],[0]))/np.dot(v1, v1)  # coefficient of projection w on v1
        w_norm = np.linalg.norm(w,\
        axis=0)  # normalized vector v, which equals sum of all coefficients ctot.
        v_a, v_b = c0/w_norm, c1/w_norm  # normalized coeficients
        return v_a, v_b

    @cached_property
    def theta(self):
        r=self.solve_equations
        a, b = abs(r.y[0][self.tx_i])**2, abs(r.y[1][self.tx_i])**2
        w = np.array([a, b])
        v_a, v_b = self.linear_projection(w)
        theta = np.arctan(v_b/v_a)
        return theta

    @cached_property
    def phi(self):
        r=self.solve_equations
        return (angle(r.y[1])-angle(r.y[0]))[self.tx_i]
```

```
def create_theta_phi_sequence(N,t_,Ng,gK,Om,gnr,\
    G12,t0,tau,gmax,phiR0,a0,b0,noise_level=1e-8):
    theta_list, phi_list = np.empty((N)), np.empty((N))
    for i in range(N):
        DMF = DensityMatrixApproximation(t_,Ng,gK,Om,gnr,\
        G12,t0,tau,gmax,noise_level,phiR0,a0,b0)
        theta_list[i], phi_list[i] = DMF.theta, DMF.phi
    return theta_list, phi_list


def plot_theta_phi_sequence(theta_list, phi_list, font_scale=6, line_width=2):
    phi_list=phi_list%(2*np.pi)
    sns.set_context("paper", font_scale=font_scale, rc={"lines.linewidth": line_width})
    fig, (ax1,ax2) = p.subplots(2, 1, figsize=(30,20))
    ax1.plot(2*theta_list, linewidth=5, color="cornflowerblue", rasterized=True)
    ax1.plot(2*theta_list, "o", color="maroon", markersize=20, rasterized=True)
    ax1.set_ylabel("Contrast $\u03B8$ (rad)")
    ax1.set_yticks([-np.pi/2,0,np.pi/2])
    ax1.set_yticklabels(["$-\pi/2$", "0", "$\pi/2$"])
    ax1.set_ylim(-np.pi/2,np.pi/2)
    ax1.set_xticks([])

    ax2.plot(phi_list, linewidth=5, color="cornflowerblue", rasterized=True)
    ax2.plot(phi_list, "o", color="maroon", markersize=20, rasterized=True)
    ax2.set_xlabel("Single-shot image #")
    ax2.set_ylabel("Relative phase $\u03C6_R$ (rad)")
    ax2.set_yticks([0,np.pi,2*np.pi])
    ax2.set_yticklabels([0,"$\pi$","2$\pi$"])
    ax2.set_ylim(0, 2*np.pi)
    fig.tight_layout()
    p.savefig("time_theta_phi_sequence.pdf", dpi=100)
```

```
"""
Initialization parameters
"""
t_=np.array([0.0001*(i+1) for i in range(60000)])
Ng=1e6
gK=2 # 1/ps
Om=0.05 # 1/ps
gnr=5 # 1/ps
G12=200 # 1/ps
t0=2 # ps
tau=0.25 # ps
gmax=1000
noise_level=1e-8
phiR0=0
a0=1.0e-6+0.0j
b0=a0*exp(1j*phiR0*2*pi)

"""
Run a single simulation, probe a,b at tx=2.5 ps.
"""
DMF = DensityMatrixApproximation(t_,Ng,gK,Om,gnr,\
G12,t0,tau,gmax,noise_level,phiR0,a0,b0)
DMF.plot_time_evolution()
print(DMF.phi, DMF.theta)

"""
Run a series of simulations - create (theta, phi) dataset
"""
theta_list, phi_list = create_theta_phi_sequence(10,t_,Ng,gK,Om,gnr,\
G12,t0,tau,gmax,phiR0,a0,b0,noise_level=1e-8)
plot_theta_phi_sequence(theta_list, phi_list)
```
